# Immunogenicity and protection mediated by dmLT and alum adjuvants for an HIV-1 vaccine

**DOI:** 10.3389/fimmu.2025.1706958

**Published:** 2026-01-21

**Authors:** Kasey Stokdyk, Anusmita Sahoo, Sailaja Gangadhara, LaTonya D. Williams, Tiffany M. Styles, Caleb A. Hellman, Lu Zhang, Ahmad J. Odeh, Shelby Flaherty, Xiaoying Shen, Mohammad Arif Rahman, Elizabeth B. Norton, Genoveffa Franchini, David Montefiori, Pamela A. Kozlowski, Georgia D. Tomaras, Rama Rao Amara

**Affiliations:** 1 Emory National Primate Research Center, Emory University, Atlanta, GA, United States; 2 Department of Microbiology and Immunology, Emory School of Medicine, Emory University, Atlanta, GA, United States; 3 Duke Center for Human Systems Immunology, Duke University School of Medicine, Durham, NC, United States; 4 Department of Surgery, Duke University Medical Center, Durham, NC, United States; 5 Animal Models and Retroviral Vaccines Section, Basic Research Laboratory, Center for Cancer Research (CCR), National Cancer Institute (NCI), National Institutes of Health (NIH), Bethesda, MD, United States; 6 Department of Microbiology and Immunology, Tulane University School of Medicine, New Orleans, LA, United States; 7 Department of Microbiology, Immunology and Parasitology, Louisiana State University Health Sciences Center, New Orleans, LA, United States

**Keywords:** adjuvant, alum, dmLT, HIV-1, non-human primates, vaccine

## Abstract

The development of an effective HIV-1 vaccine is of paramount importance to global health. Here, we compared the influence of two adjuvants, *Escherichia coli* double-mutant heat-labile toxin (dmLT) and alum, on the protective immunity induced by a cyclically permuted trimeric HIV-1 envelope gp120 protein (CycP-gp120) boost. Two groups of rhesus macaques received two modified vaccinia Ankara (MVA)/SHIV C.1086 primes followed by a CycP-gp120 protein boost adjuvanted with either dmLT (n = 9) or alum (n = 10). A group of unvaccinated macaques (n = 8) served as controls. All animals were intrarectally challenged with heterologous SHIV.CH505.375H.dCT weekly for 7 weeks. Following the challenge, dmLT-adjuvanted animals showed significant protection with a vaccine efficacy of 60.8% per exposure (*p* = 0.0246). Alum-adjuvanted animals did not show significant protection (*p* = 0.1575). Both adjuvants induced comparable envelope-specific binding antibody in serum and rectal secretions with broad V1V2 scaffold-binding specificity. IL-6 plasma concentration correlated positively with V1V2 scaffold-binding and increased after vaccination with both adjuvants. With respect to CD4 T cells, dmLT induced higher frequencies of proliferating central memory (T_CM_) and ICOS^+^ cells in blood compared to alum. However, these proliferating CD4 T_CM_ cells showed a decrease in the proportion of gut-homing receptor α4β7-expressing cells in the dmLT group compared to the alum group at week 2 post-protein boost. The V1V2 scaffold-specific IgG, proliferating T_CM_ and ICOS^+^ CD4 T-cell frequencies, and plasma IL-6 concentration associated positively with protection. These data demonstrate that the vaccine adjuvants dmLT and alum differentially modulate protective helper T-cell responses induced by the CycP-gp120 protein, highlighting the importance of an appropriate adjuvant for eliciting a protective immune response against HIV-1.

## Introduction

Nearly one quarter of the 40 million people living with HIV-1 do not receive antiretroviral treatment (ART), contributing to over 1 million new infections per year. A prophylactic HIV-1 vaccine would significantly limit these new infections in a cost-effective manner (1). However, an effective HIV-1 vaccine has remained elusive, largely due to the need for it to produce broadly neutralizing antibodies (bnAbs) and a strong CD8 T-cell response without inducing proliferation of target cells. Neutralizing antibodies prevent infection at the cellular level by binding to vulnerable sites on the HIV-1 envelope (Env) protein, including the CD4 binding site, V2, V3, and apex regions (2). They are highly effective in preventing infection by homologous HIV-1 strains. However, due to the diversity of current circulating HIV-1 strains, an effective HIV-1 vaccine that relies solely on neutralizing antibodies must elicit bnAbs. While some infected individuals have produced bnAbs to HIV-1, no HIV-1 vaccine to date has been successful in replicating them (3).

In contrast, HIV-1 vaccines have successfully elicited protective non-neutralizing antibodies (nnAbs) with certain specificities and protective antigen-specific T cells. While nnAbs do not prevent infection on a cellular level, they can clear infected cells through Fc-mediated, cell-dependent functions, including Ab-dependent cell-mediated cytotoxicity (ADCC), Ab-dependent cellular phagocytosis (ADCP), and Ab-dependent cell-mediated viral inhibition (ADCVI). Several studies in humans and non-human primates (NHPs) have shown that vaccine-induced polyfunctionality in nnAbs is associated with protection and viral control (4–11). Accordingly, vaccine effectiveness is partially determined by the Fc receptor phenotype, including immunoglobulin G (IgG) subclass and Fc glycosylation, both of which determine Fc receptor binding and subsequent functionality (4, 10, 12–15). For instance, HIV-1 Env-specific IgG3 was associated with decreased risk of HIV-1 acquisition in the RV144 trial, the only vaccine clinical trial to show some protection against HIV-1 (4, 15). Moreover, nnAbs of certain specificities are known to be protective. IgG specific to the gp70-scaffold V1V2 antigens has been positively associated with protection and viral control in both rhesus macaques and humans (8–10, 15–21).

HIV-1-specific CD8 T cells are also crucial for the clearance of infected cells and are most effective as tissue-resident memory cells (T_RM_) at the site of infection (22). The type of vaccine-induced CD4 T-cell response also plays an important role in modulating HIV-1 vaccine efficacy. HIV-1-specific T follicular helper CD4 T cells (T_FH_) are crucial for generating effective antigen-specific B-cell responses, which are associated with protection (23). T_H_1 responses are necessary to stimulate a productive CD8 T-cell response; however, our group and others have shown that an especially strong T_H_1 response in the absence of a neutralizing antibody response can diminish vaccine protection (23, 24). This is likely attributable to the high expression of CCR5 on IFNγ^+^ cells and migration to mucosal tissue, resulting in higher frequencies of HIV-1 target cells at the site of infection (23). Therefore, a protective CD4 T-cell response to HIV-1 must balance the need for CD8 T-cell and B-cell help while minimizing the generation of target cells.

Adjuvants can influence HIV-1 vaccine protection by modulating B-cell class switching, cytotoxic CD8 T-cell activation, and CD4 T-cell differentiation (25). By interacting with specific innate receptors of antigen-presenting cells (APCs), adjuvants trigger signaling pathways that determine the costimulatory signals that an APC provides to antigen-specific T helper cells. Therefore, adjuvants play a crucial role in developing the strength and quality of the subsequent adaptive immune response. We have previously described a cyclically permuted trimeric gp120 (CycP-gp120) antigen, which elicits a stronger V1V2 scaffold-binding antibody response in rabbits and rhesus macaques (RMs) than monomeric gp120 (26–28). In two separate NHP studies, we demonstrated that a modified vaccinia Ankara (MVA) prime/CycP-gp120 protein boost vaccine approach protects 40% of vaccinated animals when the protein boost is adjuvanted with double-mutant heat-labile toxin (dmLT) (10, 29). DmLT is a derivative of enterotoxigenic *Escherichia coli*’s heat-labile toxin and has primarily been used in mucosal vaccines against diarrheal diseases, including in at least 10 human trials (30–32). It is overall safe and well-tolerated in humans, with only mild side effects reported. In this study, we sought to determine if dmLT was necessary for the protective response elicited by the CycP-gp120 antigen by comparing it to alum. Alum is a well-characterized systemic adjuvant that is licensed for human use and was the adjuvant used with the AIDSVAX B/E gp120 protein boost in the RV144 trial (16, 25, 33–37). After heterologous challenge with SHIV.CH505.375H.dCT, only animals in the dmLT group were significantly protected against the acquisition of infection. The dmLT and alum adjuvants showed differential modulation of a CD4 T-cell response that was associated with protection, demonstrating the importance of an appropriate adjuvant for eliciting a protective immune response to HIV-1.

## Results

### Vaccination with dmLT but not alum provides significant protection against intrarectal SHIV.CH505 challenges

**Figure 1:**
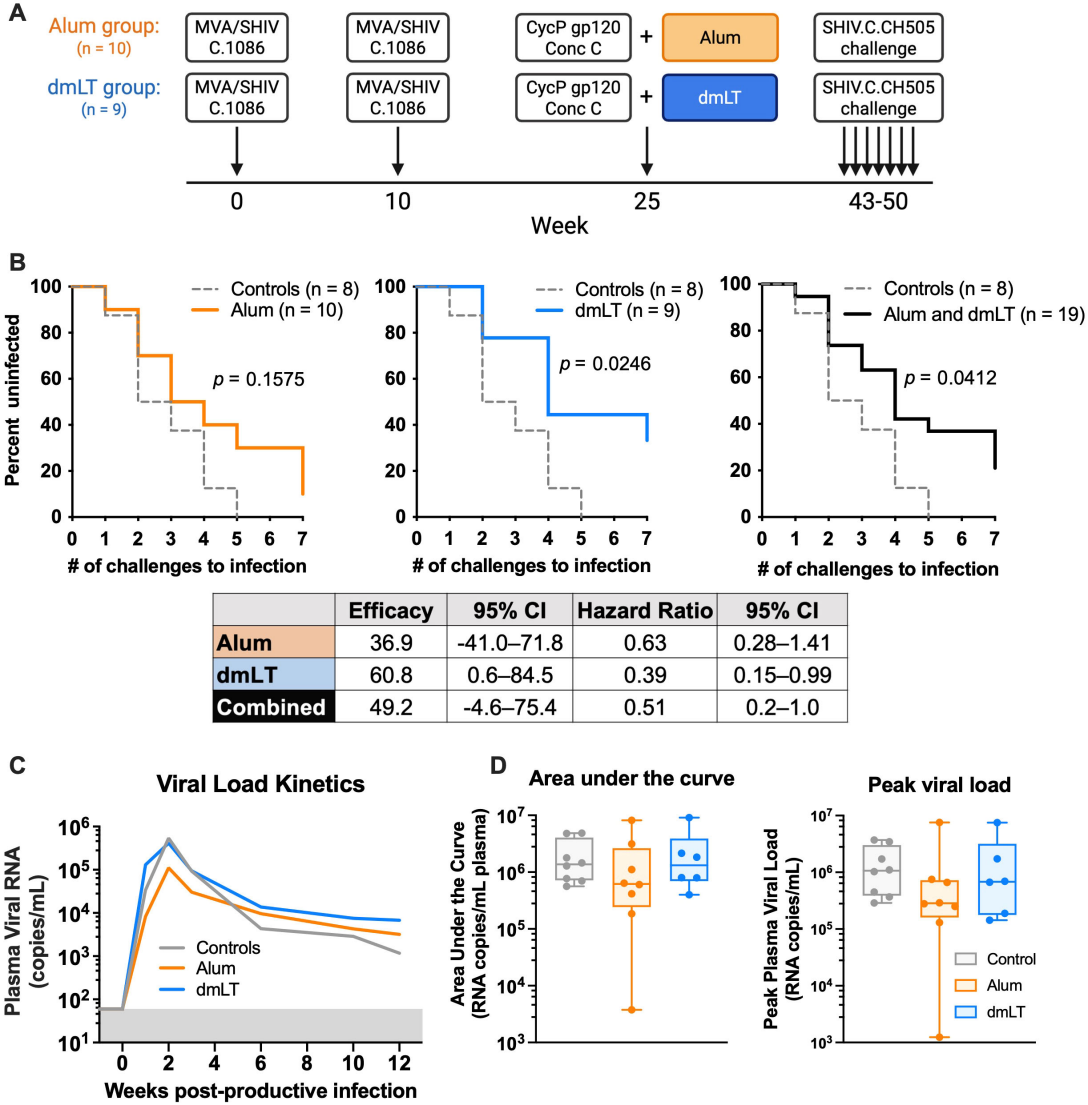
Only the double-mutant heat-labile toxin (dmLT) group was significantly protected from challenge. **(A)** Summary of the rhesus macaque (RM) HIV-1 vaccine study design. Group colors are consistent throughout figures. Modified vaccinia Ankara (MVA) expresses C.1086 Env and SIVmac239 gag, and pol. ConC is consensus C. Created using BioRender.com. **(B)** (Top) Acquisition of SHIV.CH505.375H.dCT through intrarectal challenge of unvaccinated control (n = 8), alum (n = 10), and dmLT (n = 9) groups (Kaplan–Meier infection curves, log-rank Mantel–Cox test). (Bottom) Vaccine efficacy per exposure and hazard ratio as compared to unvaccinated controls, with 95% CI. **(C)** Post-infection plasma viral load kinetics of unvaccinated control (n = 8 infected), alum (n = 9 infected), and dmLT (n = 6 infected) groups, shown by geometric mean (geomean). Gray background indicates threshold for positive viral load (n = 60 copies/mL). **(D)** Viral load area under the curve (left) and peak viral load (right) (one-way ANOVA on transformed data, Tukey’s multiple comparison follow-up). One infected animal from the alum group was excluded from area under the curve (AUC) and peak analysis due to missed sampling at week 1 post-productive infection. Box indicates 25th–75th percentile, horizontal bar indicates median, and whiskers indicate min–max.

To address the influence of the adjuvant used with a CycP-gp120 protein boost on protective immunity, we designed an NHP study to compare dmLT and alum (**Figure 1A**). First, we vaccinated 19 NHPs intradermally with previously described (10) recombinant MVA (rMVA; 10^8^ pfu per dose) expressing C.1086 gp150 and SIVmac239 Gag and Pol at 0 and 10 weeks. A clade B version of this MVA has previously been tested in phase 2a studies in humans (38, 39). Then, at 25 weeks, we boosted all animals with 100 μg consensus C (ConC) CycP-gp120 protein (10) subcutaneously. One group received protein adjuvanted with alum (1 mg dose, alum group) and the other with dmLT (5 μg dose, dmLT group). We did not observe adverse effects from any of the vaccinations. We measured antibody responses in the serum and rectal secretions, and T-cell responses in the blood and rectal tissue after each vaccination. To model intrarectal infection in men who have sex with men (MSM) and evaluate protection, at 43 weeks, we began weekly intrarectal challenges with heterologous SHIV.CH505.375H.dCT (a tier 2 clade C virus, hereafter referred to as SHIV.CH505 or challenge strain). All vaccinated animals, along with an additional group of eight unvaccinated animals (control group), were challenged until productively infected (plasma viral load >60 RNA copies/mL for two consecutive weeks), or a maximum of seven challenges.

All (eight out of eight) control animals became infected by five challenges with a rate of 35% infection per exposure (# infections/total # challenges). However, 33% (three out of nine) of animals in the dmLT group and 10% (one out of 10) of animals in the alum group remained uninfected at the end of seven challenges (**Figure 1B**). Protection (based on the rate of infection acquisition) in the dmLT group was significantly better compared to controls (*p* = 0.0246, log-rank Mantel–Cox test), with a vaccine efficacy per exposure of 60.8% (95% CI = 0.6%–84.5%). In contrast, the protection in the alum group was not significantly better than controls (*p* = 0.1575, log-rank Mantel–Cox test), and the vaccine efficacy per exposure was 36.9% (95% CI = −41% to 71.8%). When both the alum and dmLT groups were considered together, protection was also significantly better compared to controls (*p* = 0.0412, log-rank Mantel–Cox test) with an efficacy of 49% per exposure (95% CI = −4.6–75.4%; **Figure 1B**). These data demonstrate that CycP-gp120 protein adjuvanted with dmLT elicits significant protection against a heterologous clade C SHIV challenge, but CycP-gp120 with alum does not. These results also extend the protection afforded by the clade C MVA prime, CycP-gp120/dmLT boost vaccine observed in a previous study (10) to another heterologous tier 2 clade C SHIV.

We also monitored post-infection plasma viral load (PVL) to determine if vaccination contributed to better viral control. PVL peaked at week 2 and reached setpoint by week 10 in most animals (**Figure 1C**, **Supplementary Figure S1A**). The area under the curve (AUC) of PVL ranged from 3.8 × 10^3^ to 9.1 × 10^6^ RNA copies/mL but did not differ significantly between the vaccine and control groups (one-way ANOVA; **Figure 1D**). Similarly, we did not observe any significant differences in peak PVL between the groups, which ranged from 1.2 × 10^3^ to 7.6 × 10^6^ RNA copies/mL (one-way ANOVA). Taken together, animals vaccinated with dmLT adjuvant showed enhanced protection from acquisition of infection; however, those that were infected did not control the virus more efficiently than the control and alum group animals.

### Both adjuvants elicit a strong anti-Env and anti-V1V2 scaffold-binding antibody response in serum

**Figure 2:**
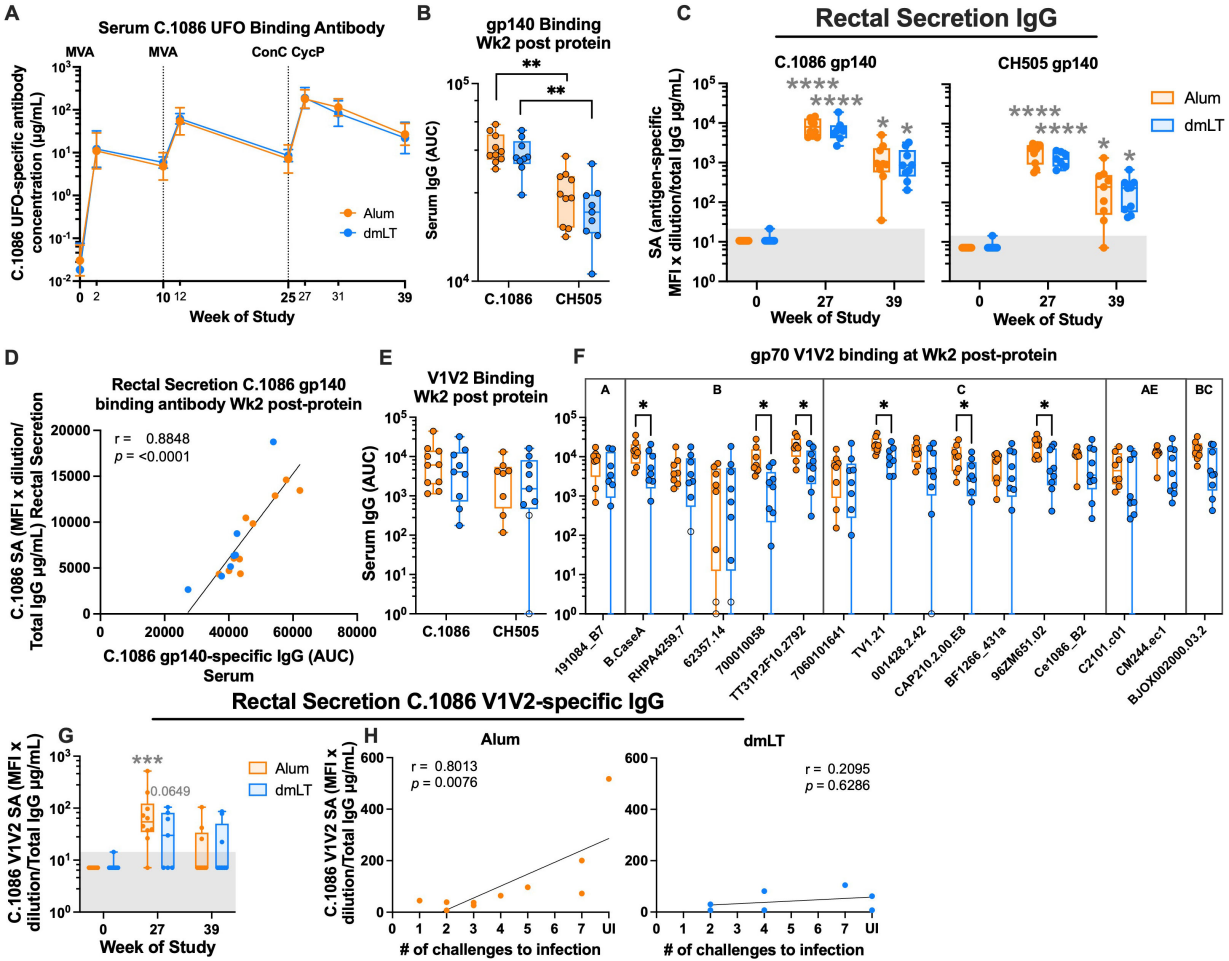
Both adjuvants elicit strong V1V2 binding. **(A)** Serum C.1086 UFO-binding IgG kinetics throughout vaccination, shown by geometric mean concentration (μg/mL). Error bars indicate geometric SD. **(B)** Serum IgG binding of C.1086 (vaccination strain) and SHIV CH505 (challenge strain) gp140 at week 2 post-protein [area under the curve (AUC)]. **(C)** Rectal secretion IgG specific activity (SA; MFI × dilution/total IgG μg/mL) for C.1086 (left) and SHIV CH505 (right) gp140 at weeks 0, 27, and 39 of the study. **(D)** Spearman’s rank correlation of serum binding antibody AUC and rectal secretion IgG SA for C.1086 gp140 at week 2 post-protein. **(E)** Serum binding to C.1086 (vaccination strain) V1V2 tags and HIV transmitted/founder (TF) CH505 (challenge strain) V1V2 scaffold at week 2 post-protein (AUC). **(F)** Serum binding to V1V2 regions from a panel of HIV-1 strains at week 2 post-protein (AUC). **(G)** Rectal secretion IgG specific activity (SA; MFI/μg/mL of IgG) for C.1086 V1V2 tags at weeks 0, 27, and 39 of the study. **(H)** Spearman’s correlation of rectal secretion C.1086 V1V2 SA at week 2 post-protein and number of challenges to infection by group. Statistics: **(B, E)** Wilcoxon test for comparisons of binding to different antigens within groups, Mann–Whitney tests for comparisons between groups. **(C, G)** Kruskal–Wallis test followed by Dunn’s multiple comparison for comparisons to baseline, Mann–Whitney for comparisons between groups. **(F)** Multiple uncorrected Mann–Whitney tests. All panels: gray stars indicate significant difference from baseline, and black stars with brackets indicate differences between groups or antigens. (B, C, E–G) Box indicates 25th–75th percentile, horizontal bar indicates median, and whiskers indicate min–max. **(C, G)** Gray background indicates antigen-specific threshold. **(E, F)** Open circles indicate animals with responses less than 3× higher than baseline. *p < 0.05, **p < 0.01, ***p < 0.001, and ****p < 0.0001.

To understand the immune correlates of vaccine protection, we determined the magnitude and specificity of antibodies in serum and rectal secretions. Serum C.1086 Env-binding IgG concentrations [measured via enzyme-linked immunosorbent assay (ELISA)] increased after the first MVA, reaching a geometric mean (geomean) of 11 μg/mL in the alum group and 12 μg/mL in the dmLT group (**Figure 2A**). The concentration increased with each boost, with a four- to fivefold increase after the second MVA, and a further three- to fourfold increase post-protein for a geomean concentration of 179 and 188 μg/mL in the alum and dmLT groups, respectively. By the pre-challenge timepoint (week 39), the C.1086 binding antibody concentration declined 6.7-fold in the alum group to a geomean concentration of 27 μg/mL and 8.6-fold in the dmLT group to a geomean concentration of 22 μg/mL. At 2 weeks post-protein, both vaccine groups showed strong binding to SHIV CH505 (challenge virus) Env gp140 as measured via binding antibody multiplex assay (BAMA), although binding to SHIV CH505 Env gp140 was significantly lower than binding to C.1086 Env gp140 in both groups (*p* alum = 0.002, *p* dmLT = 0.004, Wilcoxon test; **Figure 2B**).

We also measured Env-binding antibodies in rectal secretions at weeks 0 (baseline), 27 (2 weeks post-protein), and 39 (pre-challenge) using BAMA to track the mucosal antibody response induced after the protein boost. Following protein boost, both adjuvants elicited C.1086 Env-specific IgG in rectal secretions at 2 weeks that was significantly higher than baseline, with a median specific activity (SA; unit, MFI × dilution/total IgG μg/mL) of 7,954 in the alum group and 6,339 in the dmLT group (*p* < 0.0001 for both groups, Kruskal–Wallis test; **Figure 2C**). Binding to the immunogen strain gp140 decreased by the pre-challenge timepoint in both groups (SA median, 926 for alum and 850 for dmLT) but was still significantly higher than baseline in both groups (*p* alum = 0.021, *p* dmLT = 0.014, Kruskal–Wallis test). There was no significant difference in SA between the groups at either timepoint (Mann–Whitney test). Binding to SHIV CH505 gp140 also increased compared to baseline post-vaccination (week 27 *p* < 0.0001 for both groups, week 39 *p* alum = 0.042, week 39 *p* dmLT = 0.015, Kruskal–Wallis test; **Figure 2C**). However, binding to SHIV CH505 gp140 was four- to five-fold lower at peak (week 27) and was 3.5–5.5-fold lower at pre-challenge (week 39) than binding to C.1086. Neither vaccine induced Env-specific IgA in rectal secretions (**Supplementary Figure S2A**). There was a strong correlation between serum and rectal secretion Env-binding IgG (*p* < 0.0001; Spearman’s), suggesting that the majority of Env-specific IgG in rectal secretions is not produced locally but rather is systemic in origin (**Figure 2D**, **Supplementary Figure S2B**).

V1V2 scaffold-binding IgG has been shown to be positively associated with protection in NHPs and humans (8–10, 15–21). Therefore, we measured serum IgG reactivity to V1V2 from C.1086 and HIV CH505 Envs at the peak antibody response (week 27). We found that serum binding of C.1086 V1V2 tags (median AUC of 6,057 and 4,062 for alum and dmLT, respectively) and HIV transmitted/founder (TF) CH505 V1V2 scaffold (median AUC of 3,927 and 1,518 for alum and dmLT, respectively) was comparable between the two groups (Mann–Whitney test; **Figure 2E**). To understand cross-clade binding, we measured binding against V1V2 scaffolds from a panel of 16 HIV-1 strains representative of global HIV-1 diversity. Both protein/adjuvant boosts elicited strong V1V2 binding in the serum across all strains in the panel (**Figure 2F**). However, the titer was significantly higher in the alum group for six of the HIV-1 strains (Mann–Whitney tests). To understand V1V2-specific binding at the site of challenge, we also measured immunogen V1V2-binding antibody at the peak (week 27) and pre-challenge (week 39) in rectal secretions. Rectal secretion SA (MFI × dilution/total IgG μg/mL) to C.1086 V1V2 tags increased compared to baseline in only the alum group, for a median of 55 (*p* = 0.0005, Kruskal–Wallis test; **Figure 2G**). However, there was no statistically significant difference between the vaccination groups. By week 39, SA decreased, so there was no significant difference from baseline in either group. Interestingly, we found that the rectal secretion SA to C.1086 V1V2 at week 2 post-protein was positively correlated with protection, but only in the alum group (alum, *p* = 0.0076; dmLT, *p* = 0.6286; Spearman’s; **Figure 2H**). Similarly, V1V2 binding to Envs from several HIV-1 strains in serum and rectal secretions positively correlated with protection in the alum group, but not in the dmLT group (**Tables 1**, **2**). This suggests that the dmLT-adjuvanted vaccine was protective regardless of rectal V1V2 binding activity. Taken together, the immunogen- and challenge-specific antibody response is mostly comparable between the two groups, and the V1V2 scaffold-binding antibody is associated with protection in the alum group but not the dmLT group.

**Table 1:** Spearman’s rank correlations of antigen-specific AUC in serum with number of challenges to infection.

Serum						
Antigen	Alum			dmLT		
	r	p	Significant?	r	p	Significant?
C.1086C_V1_V2 Tags	0.50	0.15	ns	0.32	0.40	ns
gp70_CH505TF_V1V2_avi/293F	0.19	0.64	ns	0.28	0.46	ns
gp70-191084_B7 V1V2	0.25	0.55	ns	0.54	0.17	ns
gp70_B.CaseA_V1_V2	0.59	0.13	ns	0.39	0.30	ns
gp70-RHPA4259.7 V1V2	0.59	0.13	ns	0.39	0.30	ns
gp70-62357.14 V1V2	0.22	0.60	ns	0.19	0.62	ns
gp70–700010058 V1V2	0.42	0.29	ns	0.41	0.28	ns
gp70-TT31P.2F10.2792 V1V2	0.47	0.24	ns	0.19	0.62	ns
gp70–7060101641 V1V2	0.25	0.55	ns	0.31	0.41	ns
gp70-TV1.21 V1V2	0.27	0.52	ns	0.04	0.92	ns
gp70-001428.2.42 V1V2	0.73	0.05	*	0.12	0.76	ns
gp70-CAP210.2.00.E8 V1V2	0.27	0.52	ns	0.23	0.55	ns
gp70-BF1266_431a_V1V2	0.02	0.96	ns	0.42	0.25	ns
gp70-96ZM651.02 V1v2	0.68	0.07	ns	0.16	0.67	ns
gp70-Ce1086_B2 V1V2	−0.02	0.96	ns	0.28	0.46	ns
gp70-C2101.c01_V1V2	−0.42	0.29	ns	0.35	0.36	ns
gp70-CM244.ec1 V1V2	0.58	0.14	ns	0.47	0.20	ns
gp70-BJOX002000.03.2 V1V2	0.73	0.05	*	0.28	0.46	ns
Clade B V1V2 avg	0.59	0.13	ns	0.28	0.46	ns
Clade C V1V2 avg	0.61	0.12	ns	0.20	0.61	ns
Clade AE V1V2 avg	−0.11	0.80	ns	0.43	0.24	ns
ALL V1V2 avg	0.68	0.07	ns	0.39	0.30	ns

AUC, area under the curve; dmLT, double-mutant heat-labile toxin. *p < 0.05.

**Table 2:** Spearman’s rank correlations of rectal secretion antigen-specific SA (MFI × dilution/total IgG μg/mL) with number of challenges to infection.

Rectal secretions						
Antigen	Alum			dmLT		
	r	p	Significant?	r	p	Significant?
C.1086C_V1_V2 Tags	0.80	0.01	**	0.21	0.63	ns
gp70_CH505TF_V1V2_avi/293F	−0.06	0.88	ns	0.21	0.71	ns
gp70-191084_B7 V1V2	0.08	0.84	ns	0.21	0.71	ns
gp70_B.CaseA_V1_V2	0.72	0.02	*			
gp70-RHPA4259.7 V1V2	0.38	0.28	ns	−0.32	0.52	ns
gp70-62357.14 V1V2	0.67	0.04	*	0.52	0.57	ns
gp70–700010058 V1V2	0.45	0.19	ns	−0.16	0.71	ns
gp70-TT31P.2F10.2792 V1V2	0.81	0.01	**	0.08	0.86	ns
gp70–7060101641 V1V2	0.43	0.22	ns			
gp70-TV1.21 V1V2	0.41	0.24	ns	0.06	0.91	ns
gp70-001428.2.42 V1V2	0.81	0.01	**	0.08	0.86	ns
gp70-CAP210.2.00.E8 V1V2	0.45	0.20	ns	0.08	0.86	ns
gp70-BF1266_431a_V1V2	0.44	0.20	ns	0.21	0.71	ns
gp70-96ZM651.02 V1v2	0.79	0.01	*			
gp70-Ce1086_B2 V1V2	0.24	0.50	ns	0.06	0.91	ns
gp70-C2101.c01_V1V2	0.09	0.82	ns	0.34	0.48	ns
gp70-CM244.ec1 V1V2	0.46	0.18	ns	0.30	0.50	ns
gp70-BJOX002000.03.2 V1V2	0.81	0.01	**	−0.10	0.82	ns
Clade B V1V2 avg	0.76	0.01	*	0.53	0.24	ns
Clade C V1V2 avg	0.76	0.01	*	−0.13	0.79	ns
Clade AE V1V2 avg	0.43	0.22	ns	0.30	0.50	ns
ALL V1V2 avg	0.71	0.03	*	0.39	0.40	ns

SA, specific activity; MFI, mean fluorescence intensity; dmLT, double-mutant heat-labile toxin. *p < 0.05 and **p < 0.01.

### The functional profile of antibody response induced by both adjuvants is comparable

**Figure 3:**
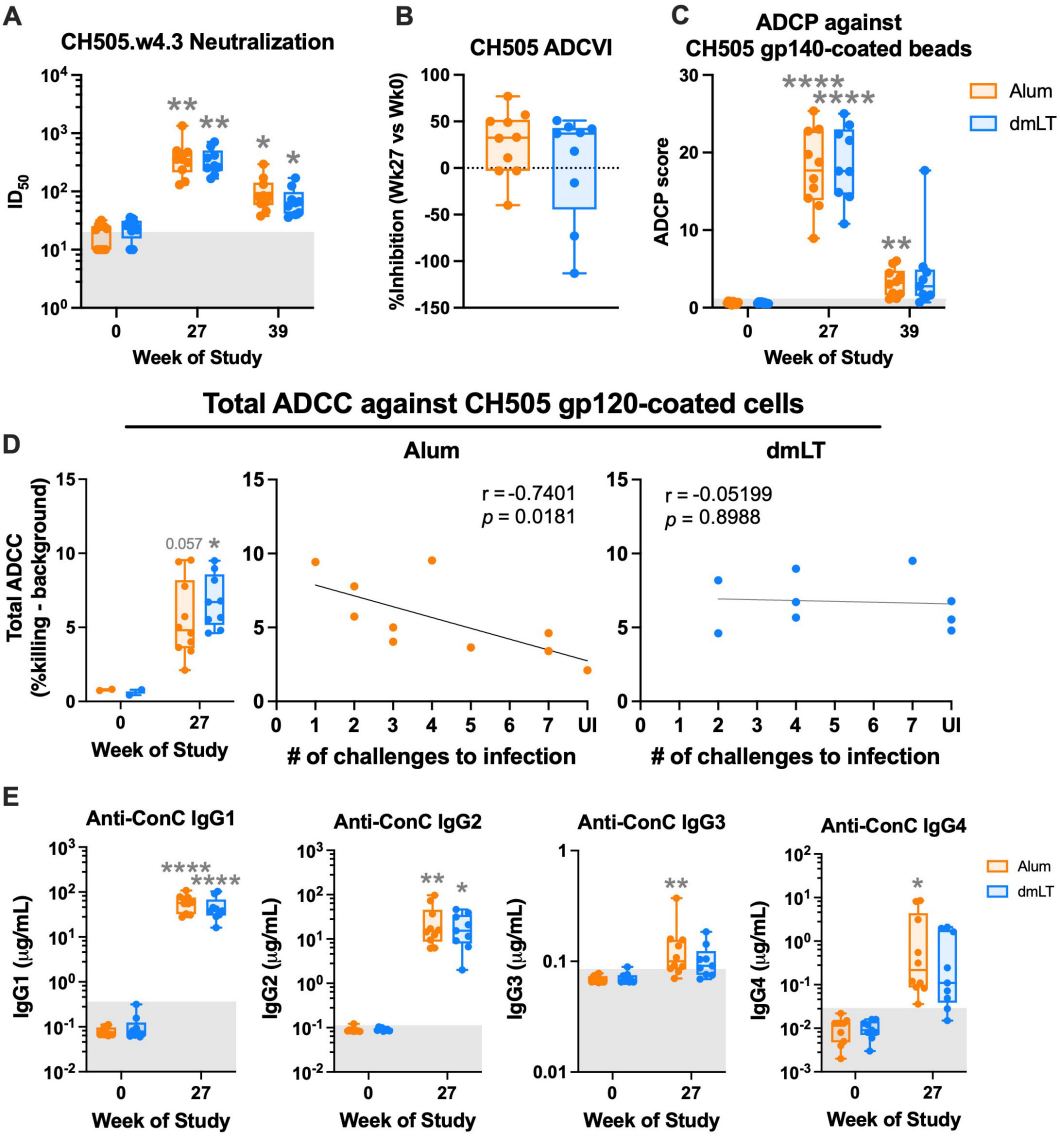
Antibody functions are comparable between vaccination groups. **(A)** Neutralization of CH505.w4.3 (tier 1) Env-pseudotyped virus at weeks 0, 27, and 39 of the study. **(B)** Antibody-dependent cell-mediated viral inhibition (ADCVI), shown by % reduction in p27 when incubated with serum from week 27 compared to week 0. **(C)** Antibody-dependent cellular phagocytosis (ADCP) score in THP-1 cells against CH505 TF Env-gp140 antigen-coated beads at weeks 0, 27, and 39 of the study. **(D)** Antibody-dependent cellular cytotoxicity (ADCC) at weeks 0 and 27 of the study (left) and Spearman’s correlation by group of ADCC at week 2 post-protein with number of challenges to infection (right). **(E)** Serum concentration of ConC CycP-gp120-specific IgG subclasses (IgG1, IgG2, IgG3, and IgG4) at weeks 0 and 27 of the study. Statistics: **(A, C)** two-way ANOVA (follow-up: Dunnett’s multiple comparison test between baseline and later timepoints, and Fisher’s LSD between groups). **(D)** Mixed-effects analysis and **(E)** two-way ANOVA, both with Fisher’s LSD follow-up. **(B)** Mann–Whitney unpaired test. All panels: gray stars indicate significant difference from baseline. **(A, B, E)** Box indicates 25th–75th percentile, horizontal bar indicates median, and whiskers indicate min–max. Gray background indicates threshold of detection **(A)** or positivity **(C, E)**. *p < 0.05, **p < 0.01, and ****p < 0.0001.

To understand how alum and dmLT influenced the function of the vaccine-induced antibody response, we measured neutralization, ADCVI, ADCP, and ADCC activities, as well as IgG subclass levels in serum at baseline, peak (week 27), and pre-challenge (week 39) timepoints. The neutralization of SHIV.CH505.w4.3 (a tier 1 virus closely related to the challenge virus) Env-pseudotyped virus increased 20-fold at 2 weeks post-protein in both vaccination groups compared to baseline (*p* alum = 0.009, *p* dmLT = 0.001, two-way ANOVA; **Figure 3A**). Neutralization activity decreased fourfold by the pre-challenge timepoint but was still measurable for most animals. There were no significant differences in neutralizing titers between the two vaccine groups. We also did not observe neutralization activity against the challenge and immunogen Envs (SHIV.C.CH505.375H and Ce1086_B2, both tier 2 Envs; **Supplementary Figure S3A**).

To further understand how the adjuvants influenced Fc-dependent effector function, we measured ADCVI, ADCP, and ADCC activities. When we measured serum ADCVI against CH505-infected CEM-NKr cells, most animals (seven in the alum group and six in the dmLT group) showed inhibition activity when comparing 2 weeks post-protein to baseline (**Figure 3B**). However, there was no significant difference in ADCVI activity between the vaccination groups (Mann–Whitney test). ADCP activity against CH505 TF gp140-coated beads increased significantly in both groups after protein vaccination (both *p* < 0.0001, two-way ANOVA) and was still measurable in most animals at the pre-challenge timepoint, but we did not observe significant differences between the groups (**Figure 3C**). Serum ADCC activity against CH505 gp120-coated EGFP-CEM-NKr-CCR5 SNAP target cells (40) significantly increased post-vaccination in the dmLT group, and there was a trend for an increase in the alum group (alum, *p* = 0.057, dmLT *p* = 0.019, mixed-effects analysis), but there was no significant difference between groups (**Figure 3D**). Unexpectedly, ADCC negatively correlated with protection in the alum group, but not in the dmLT group (alum, *p* = 0.0181; dmLT, *p* = 0.8988; Spearman’s; **Figure 3D**). We noted that the results may have been different if the assay had been conducted with SHIV-infected target cells, which we did not perform. We also measured V2-specific ADCC and observed non-significant increases in both groups and no significant difference between the groups (**Supplementary Figure S3B**). An important caveat to note is that serum from only two animals per group was tested at baseline, likely contributing to the lack of a statistically significant increase in both total and V2-specific ADCC post-vaccination.

Finally, we turned our attention to antigen-specific IgG subclass. At 2 weeks post-protein (week 27), the majority of ConC-CycP-specific IgG in both groups post-protein was IgG1, followed by IgG2, IgG4, and IgG3 (**Figure 3E**). We observed significant increases in both groups compared to baseline in IgG1 and IgG2 concentration, but IgG3 and IgG4 concentration significantly increased only in the alum group (two-way ANOVA). There were no significant differences in IgG subclass concentration between the groups. Collectively, these data demonstrate that vaccination with alum and dmLT induces comparable functional antibody responses.

### dmLT induces a higher frequency of proliferating CD4 T_CM_ cells with lower gut migration potential and higher potential for B-cell help, which are associated with protection

**Figure 4:**
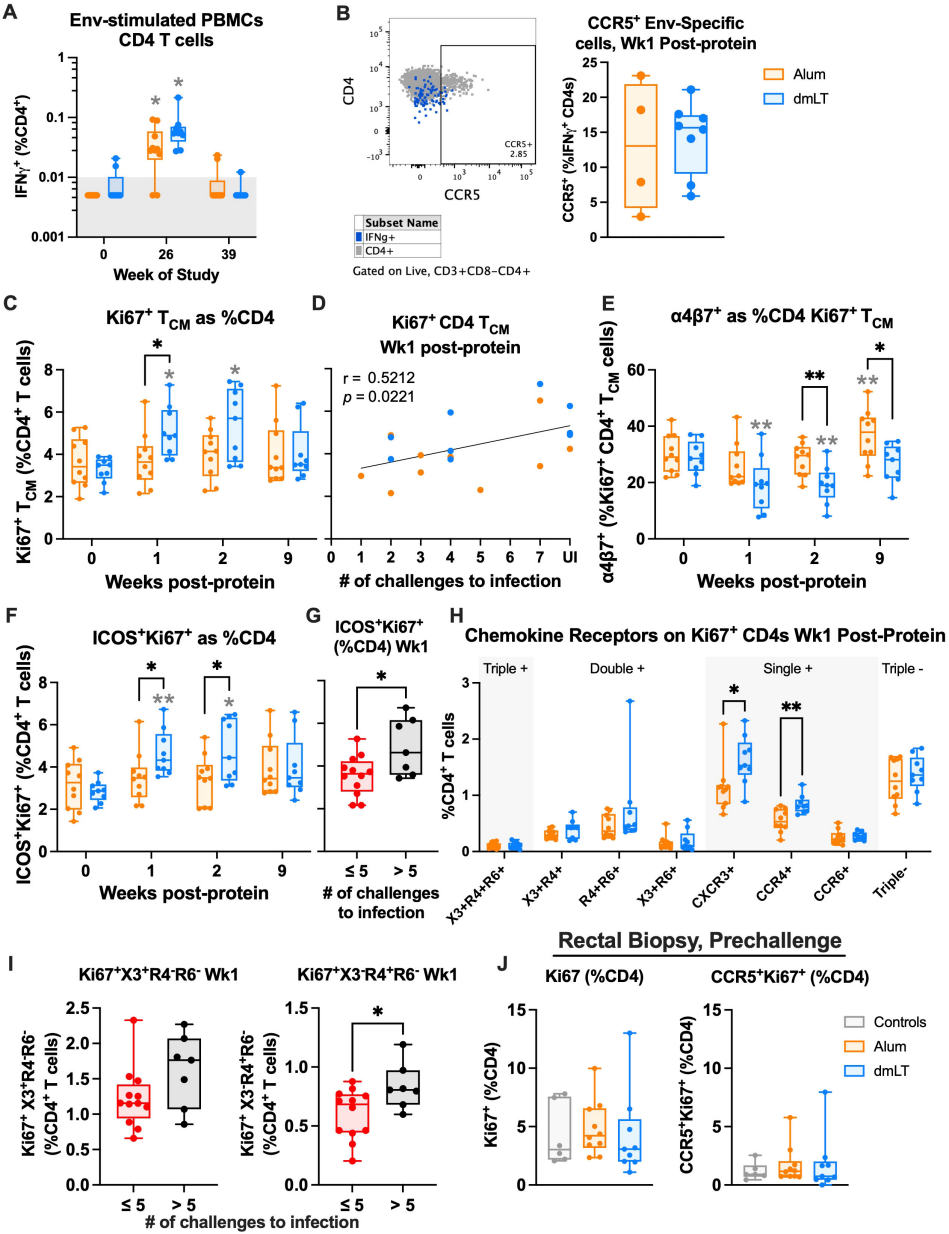
The double-mutant heat-labile toxin (dmLT) group shows higher proliferation and lower target cells compared to alum. **(A)** IFNγ^+^ as a percent of CD4 T cells in PBMCs after stimulation with Env (ICS) at weeks 0, 26, and 39 of the study. Gray background indicates threshold for positive response. **(B)** Representative (left) and quantitative (right) CCR5 frequency on Env-specific, IFNγ^+^ CD4 T cells at week 1 post-protein. **(C)** Ki67^+^ CD4 T_CM_ cells as a percent of CD4 T cells in PBMCs at weeks 0, 1, 2, and 9 post-protein, and **(D)** Spearman’s correlation of frequency at week 1 post-protein with number of challenges to infection. **(E)** α4β7^+^ as a percent of Ki67^+^ CD4 T_CM_ cells in PBMCs at weeks 0, 1, 2, and 9 post-protein. **(F)** ICOS^+^Ki67^+^ cells as a percent of CD4 T cells in PBMCs at weeks 0, 1, 2, and 9 post-protein, and **(G)** frequency at week 1 post-protein in early infected (≤5 challenges to infection) vs. late and uninfected (>5 challenges to infection) animals. **(H)** Boolean analysis of chemokine receptors on Ki67^+^ CD4 T cells at week 1 post-protein. “X3” is CXCR3, “R4” is CCR4, and “R6” is CCR6. **(I)** frequency of CXCR3 (left) and CCR4 (right) single-positive Ki67^+^ cells at week 1 post-protein in early infected vs. late and uninfected animals. **(J)** Ki67^+^ (left) and CCR5^+^Ki67^+^ as a percent of CD4 T cells in rectal biopsies at 4 weeks pre-challenge. Statistics: **(A, C, E, F)** two-way ANOVA (follow-up: Dunnett’s multiple comparison test between baseline and later timepoints, and Fisher’s LSD between groups). **(J)** One-way ANOVA (follow-up: Tukey’s multiple comparison). **(H)** Multiple uncorrected Fisher's LSD tests. **(B, G, I)** Unpaired t-test. **(A–C, E–J)** Box indicates 25th–75th percentile, horizontal bar indicates median, and whiskers indicate min–max. All panels: gray stars indicate significant difference from baseline, and black stars with brackets indicate significant difference between groups. *p < 0.05 and **p < 0.01.

To evaluate the magnitude and quality of the cellular immunity elicited by the vaccine, we performed intracellular cytokine staining (ICS) using envelope peptide pools. In the blood, MVA vaccinations induced low frequencies of IFNγ-producing CD4 and CD8 T cells in a subset of animals (**Supplementary Figures S4A, B**). This was consistent with our previous studies, where we noted that intradermal MVA vaccinations induce low frequencies of vaccine-specific T cells in NHPs (41). However, following the ConC CycP-gp120 protein vaccinations, we observed a boost of Env-specific IFNγ-producing CD4 T cells in all animals except two, and the frequency of these cells was comparable between the two adjuvanted groups (**Figure 4A**). In both groups, CD4 IFNγ^+^ frequency was significantly higher than baseline, with a median frequency of 0.03% (range, 0.005–0.09, *p* = 0.014) for alum and 0.05% (range, 0.027–0.215, *p* = 0.022) for dmLT (two-way ANOVA). By the pre-challenge timepoint, the IFNγ^+^ response returned to baseline levels, with only three animals showing a CD4 response (**Figure 4A**). The ConC CycP-gp120 vaccination did not boost IFNγ-producing CD8 T cells (**Supplementary Figure S4B**). The expression of CCR5 on Env-specific IFNγ-producing CD4 T cells was generally low and comparable between the two groups, suggesting that the majority of vaccine-induced IFNγ^+^ CD4 T cells are likely not susceptible to viral infection (**Figure 4B**). To gain insight into the antigen-specific cellular response at the site of challenge, we also performed ICS on rectal biopsies (**Supplementary Figures S4C, D**). We did not observe increases in antigen-specific CD4 and CD8 T cells compared to baseline for either group throughout the study (two-way ANOVA), suggesting that intradermal MVA and subcutaneous protein vaccinations do not induce cellular responses in the rectum.

We next defined the phenotype of proliferating (Ki67^+^) CD4 T cells post-protein vaccination, focusing on central memory (T_CM_, CD28^+^CD95^+^) frequency, α4β7 expression (potential gut-homing target cells), and ICOS^+^ (B helper potential) frequency. We used Ki67 expression post-vaccination as a proxy for antigen-specificity. The frequency of Ki67^+^ CD4 T_CM_ cells increased significantly at weeks 1 and 2 following the protein boost in the dmLT group, but not in the alum group (dmLT *p* week 1 = 0.015, *p* week 2 = 0.018, two-way ANOVA; **Figure 4C**, **Supplementary Figure S4E**). In addition, Ki67^+^ CD4 T_CM_ cell frequency at 1 week post-protein was significantly higher in the dmLT group (median of 4.9%) compared to the alum group (median of 3.6%) (*p* = 0.03, two-way ANOVA; **Figure 4C**) and positively correlated with protection (r = 0.52, *p* = 0.022; **Figure 4D**). Although we cannot be sure that these cells are antigen-specific, the proliferation of memory cells post-protein boost indicates that they are specific for Env. We also observed similar changes in total Ki67^+^ CD4 T cells (**Supplementary Figure S4F**), and animals that became infected late (required >5 challenges or protected) had higher frequencies of Ki67^+^ CD4 T cells compared to animals that became infected early (required ≤5 challenges to infection) (*p* = 0.028, t-test; **Supplementary Figure S4G**). The proportion of Ki67^+^ CD4 T_CM_ cells expressing α4β7 significantly decreased from a median of 28.5% on the day of protein boost to 19.4% at 1 week post-vaccination in the dmLT group, but there was no significant change in the alum group (dmLT *p* = 0.003, two-way ANOVA; **Figure 4E**). This decreased frequency in the dmLT group persisted at week 2 (median of 19.0%, *p* = 0.006), resulting in a significantly lower frequency than the alum group, which had a median frequency of 29.6% (*p* = 0.007, two-way ANOVA). Similar results were also observed for total Ki67^+^ CD4 T cells (**Supplementary Figure S4H**). We did not observe a difference in antigen-specific IFNγ^+^ cells (**Supplementary Figure S4I**); however, this is not entirely surprising because overall expression is lower on antigen-stimulated cells, and in our experience, ICS is relatively less sensitive and would not capture the totality of antigen-specific cells. These data collectively demonstrate that a higher frequency of proliferating CD4 T cells responded to the vaccine in the dmLT group, but mostly without gut-homing potential.

In contrast to α4β7^+^ cells, the frequency of ICOS^+^Ki67^+^ CD4 T cells increased in the dmLT group (median frequency of 4.3%) but not the alum group at 1 week following protein boost (*p* = 0.005, two-way ANOVA; **Figure 4F**). The frequency was also significantly higher compared to the alum group (median, 3.5%, *p* = 0.041, two-way ANOVA). The elevated frequency in the dmLT group persisted at week 2 post-protein (*p* = 0.010 compared to baseline and 0.040 compared to alum, two-way ANOVA). Further, animals that became infected later (>5 challenges) had significantly higher ICOS^+^Ki67^+^ frequency at 1 week post-protein (*p* = 0.019, t-test; **Figure 4G**). This is consistent with previous studies that have shown that CD4-mediated B-cell help is associated with protection (23, 42, 43). These results demonstrate that the two adjuvants induced a qualitatively different proliferating CD4 T-cell response following protein vaccination, with dmLT inducing CD4 T cells with less potential to migrate to the gut and better B helper function compared to alum.

We also used Boolean analysis of chemokine receptors to measure T_H_ differentiation on proliferating CD4 T cells and understand the impact on protection. At 1 week post-protein, both CXCR3^+^CCR4^−^CCR6^−^ (T_H_1-like) Ki67^+^ cell frequency and CXCR3^−^CCR4^+^CCR6^−^ (T_H_2-like) Ki67^+^ cell frequency were elevated in the dmLT group compared to the alum group (*p* = 0.03 and 0.004, respectively, Fisher’s Least Significant Difference (LSD) test; **Figure 4H**). The frequencies of these cells were also elevated compared to baseline in the dmLT group (**Supplementary Figure S4J**). Interestingly, although the Ki67^+^ CXCR3 single-positive cell frequency was not associated with protection, the Ki67^+^ CCR4 single-positive frequency was significantly higher in late-infected and protected animals (*p* = 0.027, t-test; **Figure 4I**). This suggests that a stronger T_H_1 phenotype is not as protective as a T_H_2 phenotype.

Finally, we measured the CD4 T-cell phenotype in rectal biopsies at the pre-challenge timepoint to understand if vaccination influenced these cells at the site of challenge and thereby influenced protection. Among CD4 T cells, Ki67^+^ and Ki67^+^CCR5^+^ frequencies did not vary significantly by vaccination group and were also not significantly different from unvaccinated controls (one-way ANOVA; **Figure 4J**). We also did not observe any significant difference in Ki67^+^α4β7^+^ or Ki67^+^CCR5^+^α4β7^+^ CD4 T-cell frequencies (**Supplementary Figures S5A, B**), total CD4 T_RM_ (CD69^+^CD103^+^) cells, or CD4 T_RM_ targets (**Supplementary Figures S5C–F**), nor Ki67^+^ or T_RM_ CD8 T-cell responses (**Supplementary Figures S5G, H**). This further supports our observations for CCR5^+^ antigen-specific CD4 T cells in blood (**Figure 4B**) and suggests that vaccination does not increase target cells at the site of challenge despite systemic CD4 T cell proliferation elicited by dmLT vaccination.

### Lower innate activation and higher IL-6 concentration are associated with better protection

**Figure 5:**
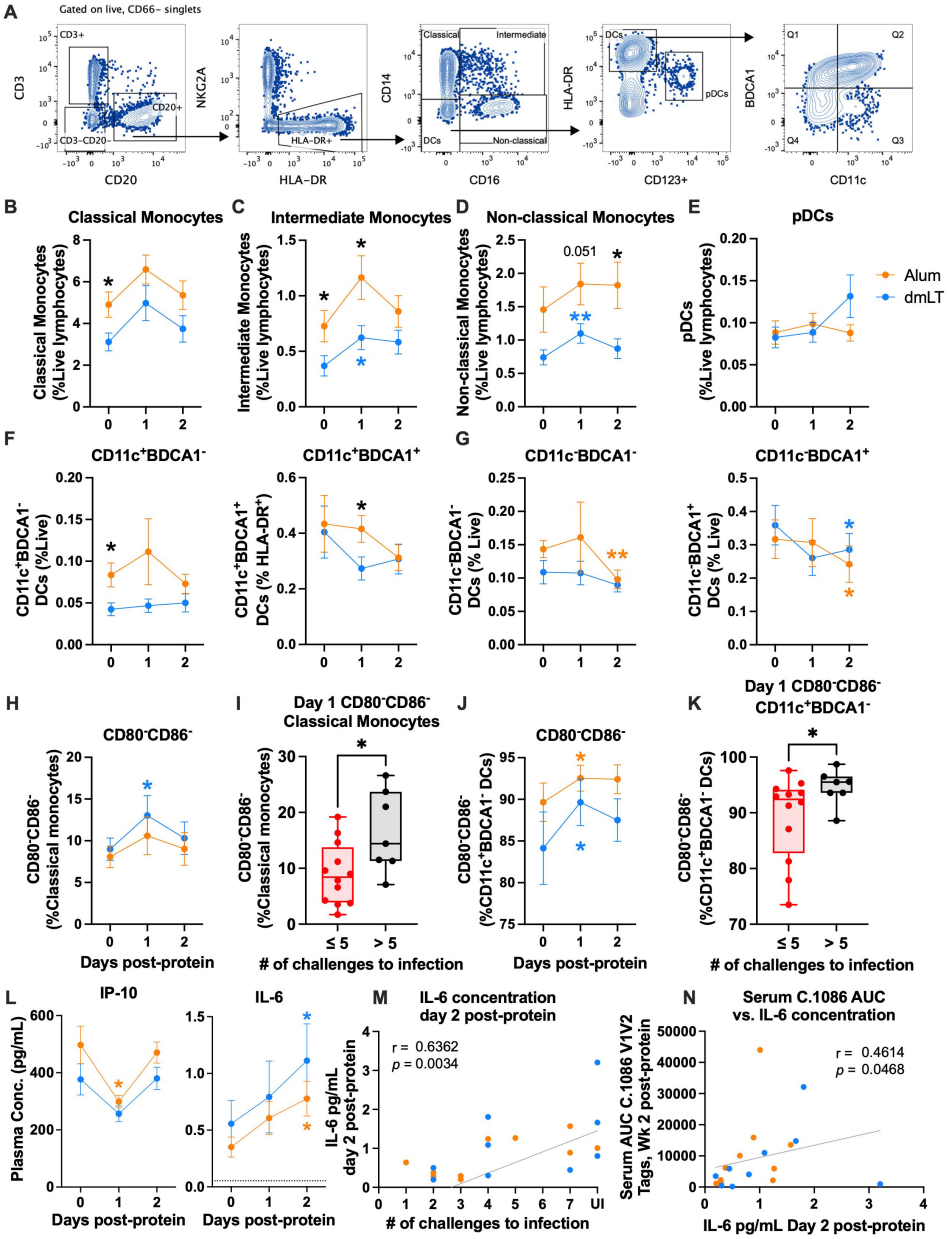
IL-6 increases in both groups and is associated with protection. **(A)** Representative flow plots for innate cell gating. **(B–E)** Mean frequency of classical, intermediate, and non-classical monocytes and plasmacytoid dendritic cells (pDCs) as a percent of live lymphocytes at days 0, 1, and 2 post-protein. **(F, G)** Mean frequency of BDCA1^−^ (left) and BDCA1^+^ (right) CD11c^+^**(F)** and CD11c^−^**(G)** DCs as a percent of live lymphocytes at days 0, 1, and 2 post-protein. **(H–K)** Mean frequency of CD80^−^CD86^−^ as a percent of **(H)** classical monocytes and **(J)** CD11c^+^ BDCA1^−^ DCs at days 0, 1, and 2 post-protein, and **(I, K)** frequency at day 1 post-protein in early infected (≤5 challenges to infection) vs. late and uninfected (>5 challenges to infection) animals. **(L)** IP-10 (left) and IL-6 (right) mean plasma concentration at days 0, 1, and 2 post-protein. **(M, N)** Spearman’s correlations of IL-6 plasma concentration at day 2 post-protein with **(M)** number of challenges to infection and **(N)** serum C.1086 V1V2 tags binding at week 2 post-protein. Statistics: **(B–H, J, L)**: two-way ANOVA (follow-up: Dunnett’s multiple comparison test between baseline and later timepoints, and Fisher’s LSD between groups). Error bars indicate SEM. **(I, K)** Unpaired t-test. Dataset in K did not meet the equivalent variance requirement, so Welch’s correction was used. Box indicates 25th–75th percentile, horizontal bar indicates median, and whiskers indicate min–max. All panels: orange and blue stars indicate significant difference from baseline within alum or double-mutant heat-labile toxin (dmLT) (respectively), and black stars indicate significant difference between groups. *p < 0.05 and **p < 0.01.

The activation of innate cells modulates the quality and function of the adaptive immunity, and very little is known about the innate activation phenotype elicited by alum and dmLT adjuvants in NHPs. To understand if differences in the activation of innate cells contributed to a difference in protection, we used flow cytometry to measure the innate immune activation in the days following vaccination with CycP-gp120 adjuvanted with dmLT or alum (**Figure 5A**). Specifically, we monitored classical (CD14^+^CD16^−^), intermediate (CD14^+^CD16^+^), and non-classical (CD14^−^CD16^+^) monocytes, plasmacytoid dendritic cells (DCs) (pDCs; CD123^+^), myeloid DCs (mDCs; CD11c^+^HLA-DR^hi^), and CD11c^−^ DCs. Among the mDCs and CD11c^−^ DCs, we also measured BDCA1 expression. All monocytes and DC subsets were found to be BDCA3^+^ (data not shown), so this marker was not considered for analysis.

On the day of protein vaccinations (day 0), the alum group had significantly higher frequencies of classical and intermediate monocytes, and BDCA1^−^ mDCs compared to the dmLT group (*p* = 0.029, 0.05, and 0.02, respectively, two-way ANOVA; **Figures 5B, C**). This could be a potential confounding factor, and results should be interpreted with caution. The significantly higher frequency in the alum group persisted at day 1 for intermediate monocytes (*p* = 0.031, two-way ANOVA; **Figure 5C**). While classical, intermediate, and non-classical monocytes all increased in both groups at day 1 post-protein, the changes were only significant in the dmLT group for intermediate and non-classical monocytes (*p* = 0.014, 0.003, respectively, two-way ANOVA; **Figures 5B–D**). At day 2, the alum group had significantly higher frequencies of non-classical monocytes than the dmLT group (*p* = 0.027, two-way ANOVA; **Figure 5D**). Overall, these data demonstrate that both adjuvants caused small increases in monocyte frequencies at day 1, with significant increases in the dmLT group for intermediate and non-classical monocyte frequencies.

Among the DC subsets, pDC and mDC frequencies remained relatively stable after vaccination (**Figures 5E, F**). However, BDCA1^+^ mDC frequency was significantly higher in the alum group at day 1 (*p* = 0.04, two-way ANOVA; **Figure 5F**), although this was due to a non-significant decrease in the dmLT group rather than an increase in the alum group. In CD11c^−^ DCs, BDCA1^−^ frequency decreased significantly in the alum group at day 2 (*p* = 0.007, two-way ANOVA; **Figure 5G**), and BDCA1^+^ frequency decreased significantly in both groups at day 2 (*p* alum = 0.027, *p* dmLT = 0.02, two-way ANOVA; **Figure 5G**).

We also measured changes in the activation of innate cells based on CD80 and CD86 expression post-protein vaccination and found that the frequency of CD80^−^CD86^−^ classical monocytes increased significantly at day 1 in the dmLT group (*p* = 0.04, two-way ANOVA; **Figure 5H**). Interestingly, uninfected and late-infected animals also had a significantly higher CD80^−^CD86^−^ classical monocyte frequency at day 1 than in animals that were infected early (*p* = 0.028, t-test; **Figure 5I**). In intermediate monocytes, non-classical monocytes, and pDCs, CD80 and CD86 expression remained relatively stable post-vaccination (**Supplementary Figures S6A–C**). However, CD86^+^ frequency decreased significantly in most DC subsets (**Supplementary Figures S6D–H**). This was often mirrored by a significant increase in CD80^−^CD86^−^ frequency in one or both vaccination groups, including in BDCA1^−^ mDCs (*p* alum = 0.014, *p* dmLT = 0.039, two-way ANOVA; **Figure 5J**, **Supplementary Figures S6F–H)**). Similar to classical monocytes, uninfected and late-infected animals had higher frequencies of CD80^−^CD86^−^ BDCA1^−^ mDCs at day 1 post-protein (*p* = 0.045, t-test with Welch’s correction; **Figure 5K**). Activation marker expression returned to baseline levels by day 2 for most innate cell subsets.

Finally, we measured plasma cytokine concentrations using Meso Scale Discovery (MSD) at innate timepoints to understand how vaccination affected innate cytokine production. Interestingly, we observed decreases in multiple inflammatory cytokines at day 1 post-protein, including IP-10, MCP-1, and MIP-1α (two-way ANOVA; **Figure 5L**, **Supplementary Figure S6I**). However, most cytokines returned to baseline levels by day 2. This decrease and return to baseline could be related to the reduction in activation marker expression on innate cells. A notable exception to these observations was IL-6, which increased ~2-fold in both groups at day 2 post-protein (*p* alum = 0.016, *p* dmLT = 0.026, two-way ANOVA; **Figure 5L**). Interestingly, the plasma IL-6 concentration at day 2 post-protein correlated positively with protection (r = 0.64, *p* = 0.0034, Spearman’s; **Figure 5M**) as well as C.1086 V1V2 binding antibody in serum at 2 weeks post-protein (r = 0.46, *p* = 0.0468, Spearman’s, **Figure 5N**). Overall, these results demonstrated a decrease in the activation of several innate cell subsets and the concentration of several inflammatory cytokines and an increase in the concentration of IL-6, a B-cell growth factor (44–46).

## Discussion

Adjuvants can modulate the innate and adaptive immunity induced by vaccination and thereby influence vaccine efficacy. Here, we investigated the influence of the vaccine adjuvants dmLT and alum on the immunogenicity and efficacy of an HIV-1 vaccine. We used a vaccination approach (MVA/CycP-gp120) that previously showed protection in two studies against a heterologous clade B and clade C SHIV challenge in the absence of an autologous neutralizing antibody response (10, 29). In the current study, we directly compared dmLT, the adjuvant used in the previous studies, with alum, which was used in the RV144 efficacy trial and is licensed for human use (16). We found that only the CycP-gp120 protein adjuvanted with dmLT significantly delays viral acquisition against SHIV.C.CH505, extending our previous findings to another clade C virus. This is significant, as clade C viruses represent the majority of the global HIV-1 health burden (47). While some animals receiving protein with alum were protected, this group was not significantly protected.

**Figure 6:**
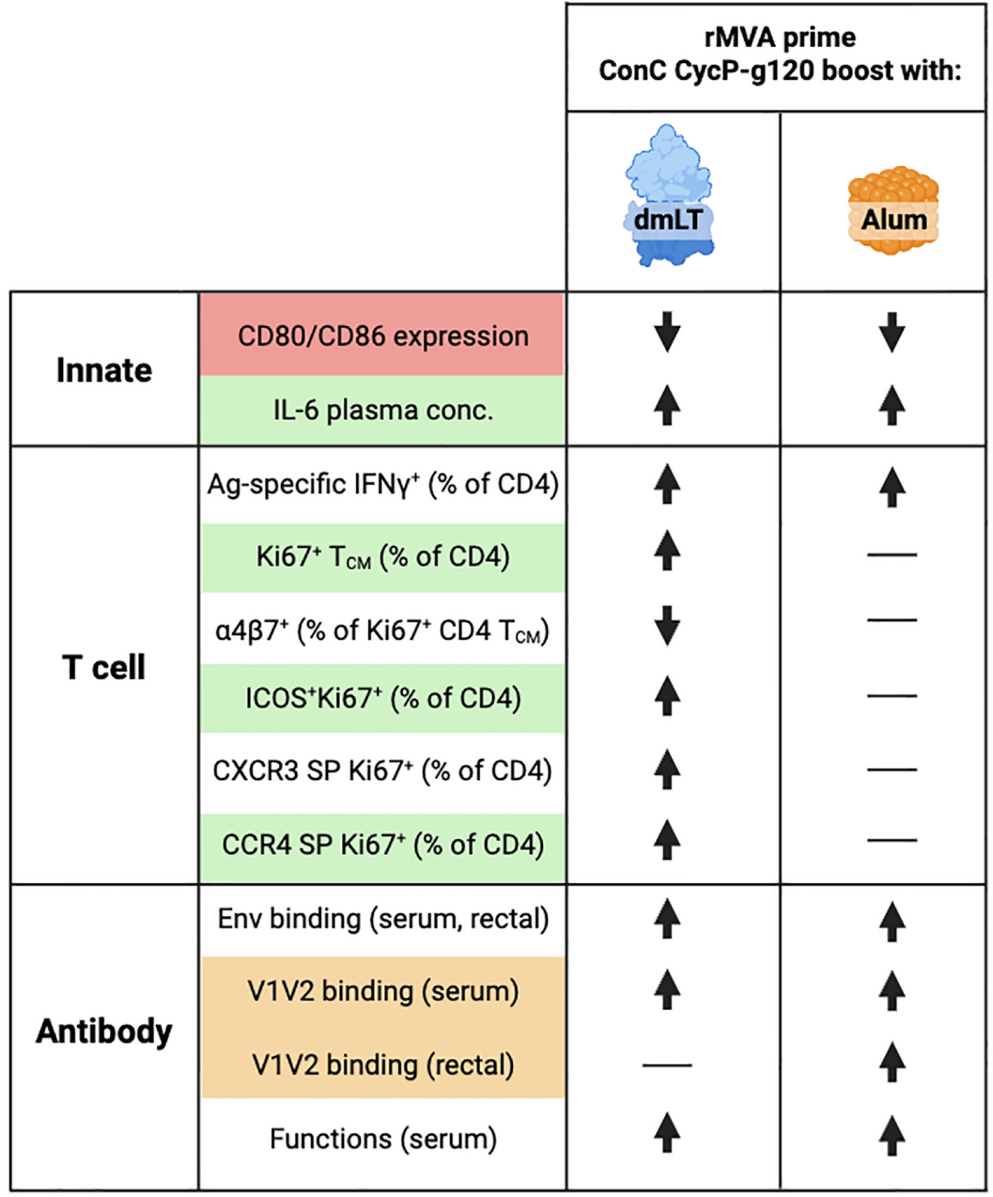
Schematic of different responses elicited by double-mutant heat-labile toxin (dmLT) and alum. Summary of innate, T-cell, and antibody responses after vaccination with ConC CycP-gp120 adjuvanted with either dmLT or alum. Arrows pointing upward indicate an increased response, arrows pointing downward indicate a decreased response, and horizontal lines (—) indicate no change from baseline. Shading indicates a correlation with protection; red indicates a negative correlation, green indicates a positive correlation, and orange indicates a positive correlation in only the alum group. T-cell responses are all in PBMCs or whole blood. “SP” stands for chemokine single-positive (Boolean analysis). Created using BioRender.com.

Our study identified a few immune responses that associate positively with protection (**Figure 6**). The dmLT group had significantly higher proliferating CD4 T_CM_ cell frequency, which was associated with protection. A CD4 memory response is crucial for an effective vaccine response due to its promotion of CD8 T- and B-cell responses; however, in the context of HIV-1, proliferating CD4s can be negatively associated with protection due to the potential increase in target cells. In the current study, the positive association with protection could be explained by the decrease in α4β7^+^ frequency within this population, which only occurred in the dmLT group and is consistent with our previous study (10). Total and memory CD4 T cells expressing a4β7 have been shown to be preferentially infected and depleted in both NHPs and humans (48–52), highlighting the potential for α4β7^+^ cells to be targets. Therefore, we hypothesized that lower α4β7 expression on proliferating CD4 T_CM_ cells could have reduced homing of potential target cells to the mucosa for the dmLT group. Carnathan et al. showed that rectal biopsy frequencies of activated CCR5^+^ CD4 T cells associated with infection and higher early viral load (24). Additionally, we have shown that high frequencies (>1%) of antigen-specific IFNγ^+^ CD4 T cells in the blood post-vaccination are associated with high frequencies of IFNγ^+^ CD4 in the mucosa at memory timepoints as well as loss of protection (23). Therefore, we measured frequencies of total activated (CCR5^+^Ki67^+^) CD4 T cells and antigen-specific IFNγ^+^ CD4 T cells in the mucosa. We did not observe significant differences between controls, alum, and dmLT for either of these parameters, and peripheral IFNγ^+^ CD4 T cells were very low post-protein (median of 0.05%), which was in alignment with the very low IFNγ response in the rectum. Therefore, we do not expect that IFNγ^+^ CD4s played a significant role in differential protection. That the stronger systemic response did not elicit more mucosal target cells in the dmLT group compared to the control and alum groups is encouraging and is possibly attributable to lower α4β7 frequency on the proliferating cells. It is also possible that lower α4β7 in the dmLT group could have resulted in key phenotypic differences at the mucosa that we did not measure. First, we unfortunately did not measure CCR5^+^HLA-DR^+^ CD4 T cells in the rectum, which were uniquely associated with infection in the Carnathan study, while CCR5^+^Ki67^+^ CD4 frequency was associated with higher early plasma viremia. Second, in our experience with mucosal cells, the ICS assay does not capture the majority of antigen-specific cells, which are more likely to be infected than non-antigen-specific cells. Future studies should investigate whether either or both of these target cells are reduced after vaccination with dmLT.

ICOS^+^Ki67^+^ frequency was also significantly higher at 1 week post-protein in late-infected animals and the dmLT group. This could indicate that vaccination with dmLT produces a CD4 T-cell response with more potential to help B cells, although it is important to note that we did not observe stronger antibody responses in the dmLT group post-protein compared to alum. It is possible that the ICOS-expressing cells mediated other helpful B-cell responses that we did not measure, for example, plasmablast frequencies, bone marrow plasma cell frequencies, or memory cell frequency. Additionally, ICOS is known to be involved in other T helper responses, including T_H_1 and T_H_2 differentiation (53). In alignment with this, we found that dmLT induced higher frequencies of proliferating CXCR3 single-positive (T_H_1-like) and CCR4 single-positive (T_H_2-like) cells (**Figure 4I**). Only the CCR4^+^ frequencies were higher in protected and late-infected animals, consistent with our previous finding that disproportionate T_H_1 responses are negatively associated with protection (23). This promotion of several types of differentiation is in alignment with literature, where it has been shown that dmLT induces a mixture of T_H_1/2/17 responses *in vitro* (54, 55) and *in vivo* (56–59), with the proportional strengths varying depending on the antigen, route, and model organism. Collectively, the use of dmLT with ConC CycP-gp120 promotes several T helper responses correlated with protection. These responses may be related, but further testing is needed to corroborate this.

There are multiple differences by which alum and dmLT activate innate immune cells. When dmLT is internalized in APCs, the A subunit adds ADP-ribose to Gsα, which activates adenylate cyclase and increases intracellular cyclic AMP (cAMP) (31). This activates several transcription factors, including PKA and CREB (55, 60). Independent of PKA, dmLT has also been shown to activate the inflammasome and subsequently, caspase-1 (55, 58, 60, 61). Activated DCs then release cytokines, including IL-1β, IL-23, and IL-6 (55, 61). In contrast, the mechanism by which alum activates innate cells to promote immune responses is still under debate despite its long-standing use (25). Studies have shown that alum largely acts independently from TLR signaling and NFκB (62, 63). Like dmLT, alum also induces the production of IL-1β and IL-6 in an NLRP3 inflammasome-dependent manner *in vitro*; however, *in vivo* studies present conflicting evidence on the relevance of this pathway (64–69). Other studies have suggested that alum also acts through the release of uric acid and extracellular DNA (70–72). Despite these different mechanisms, we did not observe any differences between the two groups in innate responses that were associated with protection. CycP-gp120 adjuvanted with alum and dmLT elicited similar reductions in activation markers on innate cells, which were associated with protection. This finding was surprising; however, because this analysis was conducted on peripheral blood mononuclear cells (PBMCs), it is possible that we measured a migration of activation marker-expressing cells out of the blood and into lymph nodes rather than a reduction in the expression of activation markers on these cell types. That we cannot differentiate between the two possibilities is a limitation of the study. In contrast, the simultaneous reduction in plasma concentration of many pro-inflammatory cytokines suggests that there could have been an overall reduced inflammatory state at day 1 that largely normalized by day 2. IL-6 was the sole exception to the observed reductions in inflammatory markers at innate timepoints. Consistent with their known mechanisms, vaccination with both alum and dmLT resulted in increased IL-6 plasma concentration over days 1 and 2 post-boost. Because this was performed on plasma, we cannot determine which cells are producing IL-6, although the early timing post-protein suggests innate cells as a source. The increase in IL-6 would temporally precede germinal center (GC) formation even in a recall response (73). Therefore, it is possible that a downstream effect of elevated IL-6 could be the promotion of B-cell responses. This is supported by the observed association with the V1V2-specific antibody response (**Figure 5N**), as well as the literature (44–46, 74), but further testing is needed to determine the relationship beyond correlation.

Surprisingly, alum outperformed dmLT in several measurements of IgG V1V2 binding. V1V2-binding antibody in the serum was significantly stronger for six out of the 16 strains we measured in the alum group, and only the alum group experienced an increase in immunogen-strain V1V2 tag binding in rectal secretions post-vaccination. These responses correlated with better protection, although only in the alum group. This is consistent with our past studies, where we observed a trend of challenge strain V2-binding being associated with protection and a significant inverse correlation with peak viral load (10, 29). The lack of correlation between V1V2 binding and protection in the dmLT group suggests that other responses, likely CD4 T-cell responses, are playing a stronger role in protection for this group, although further testing is needed to determine causation. The advantage in V1V2 binding in the alum group could be explained by alum’s formation of “depots” at the site of immunization that slowly release antigen (75–77). More recently, the slow release of antigen has been demonstrated to elicit strong germinal center and antibody responses (78–81). Collectively, these observations suggest that protection induced by alum may be improved by employing additional CycP-gp120 protein boosts with alum. Indeed, a recent human HIV-1 vaccine efficacy trial (HVTN 107) showed that protective V1V2 responses increased in magnitude and breadth with further boosts of protein adjuvanted with alum (82). We did not consider two protein boosts in the current study since we have previously shown that repeated exposure to dmLT elicits anti-dmLT antibodies that result in a blunted antibody response to the immunogen (29). Future studies should investigate whether additional boosts of CycP-gp120 adjuvanted with alum could increase protection via boosting of V1V2-specific activity at the site of challenge.

Several limitations should be considered when interpreting our findings. First, our study is not mechanistic in nature. Although we identified several interesting correlates of protection, we cannot determine if any of them are causative. Therefore, all potential mechanisms for protection that we propose here are only hypotheses that would require further testing. Second, because of the cost limitations of NHP studies, we could only test one dose for alum and dmLT (1 mg and 5 μg, respectively). We chose the alum dose based on what is used in humans, and the dmLT dose based on our previous study (10). However, because the doses were different, we cannot rule out the possibility that the difference in protection could be due to differences in adjuvant dose. Future studies could compare multiple doses of each adjuvant to address this. Finally, we did not measure several immune responses, including ADCC with SHIV-infected targets, Fcγ receptor binding profiles, rectal biopsy CCR5^+^HLA-DR^+^ CD4 T cells, and B-cell clonality. Future directions should also consider these responses to determine if they were different between the groups or correlated with protection.

Overall, CycP-gp120 vaccination with dmLT but not alum protected animals against intrarectal SHIV.CH505 challenge. Protection was associated with proliferating CD4 T_CM_ cells, CD4 T cells with B helper potential, and IL-6 levels in plasma soon after protein boost. V1V2-binding IgG in the serum and rectal secretions was positively associated with protection only in the alum group, and it is possible that additional boosts of the alum-adjuvanted protein could have increased protection. The adjuvants’ differential modulation of the T-cell response and associated protection emphasize the importance of adjuvant selection in HIV-1 vaccination.

## Materials and methods

### Study design

Twenty male Indian RMs (*Macaca mulatta*) (4.6–5.1 years, mean = 4.9 years; 6.6–12.3 kg, mean = 9.17 kg) were vaccinated intradermally in the inner thigh with previously described rMVA (10), which expressed C.1086 gp150 and SIV mac239 Gag and Pol at 0 and 10 weeks, respectively (10^8^ pfu per dose). The RMs were boosted at 25 weeks with 100 μg ConC CycP-gp120 protein (described previously (10)) adjuvanted with either alum (1 mg dose, n = 10) or dmLT (5 μg dose, n = 10) subcutaneously in the inner thigh. At 18 weeks post-protein boost, weekly challenges began in which 1 mL of SHIV.CH505.375H.dCT (provided by George Shaw and Hui Li, GenBank accession #KU958487 (83),) diluted 1:250 was administered intrarectally to all vaccinated animals and 10 male unvaccinated RMs (3.8–4.4 years, mean = 3.9 years, 5.2–7.7 kg, mean = 6.55 kg). PVL for all animals was measured weekly using quantitative reverse transcriptase polymerase chain reaction (qRT-PCR) as described previously (84). Animals were challenged until they had a measurable PVL (>60 RNA copies/mL) for two consecutive weeks, or a maximum of seven challenges. PVL was also measured at 6, 10, and 12 weeks post-infection to monitor peak PVL and setpoint. All immunizations, sample collections, and challenges were performed under anesthesia by trained and study group-blinded research and veterinary staff. Veterinary staff also routinely monitored animals post-vaccination for reactogenicity and adverse effects. None were observed in the current study.

We retroactively discovered that three animals (two in the control group and one in the dmLT group) were positive for the Mamu-A*01 allele. These three animals were excluded from analysis because we found in a previous study (85) as well as a parallel study (**Supplementary Figure S7A**) that this allele can contribute to protection in the unvaccinated control animals. Therefore, the presence of two animals in the control group and one in the dmLT group with the Mamu-A*01 allele would be a confounding factor for protection in both groups. When Mamu-A*01 animals were included, the dmLT group was not significantly protected, although there was a strong trend (*p* = 0.051; **Supplementary Figure S7B**), and the alum group was not protected (*p* = 0.49; **Supplementary Figure S7B**). None of the animals were positive for Mamu-B*08 and Mamu-B*17.

### Ethics statement

All experiments involving RMs were conducted at Emory National Primate Research Center in compliance with Emory University Institutional Animal Care and Use Committee protocol 201900129 and amendment 202300645, under the U.S. Department of Agriculture regulations and Guide for the Care and Use of Laboratory Animals guidelines. Emory National Primate Research Center is accredited by the American Association for Accreditation of Laboratory Animal Care (AAALAC).

### Assays to characterize antigen-specific antibody in serum and rectal secretions

Immunogen trimer-specific IgG was quantified using ELISA. Briefly, blocking buffer [5% milk powder in 4% whey buffer with phosphate-buffered saline (PBS)-tween (PBST)] was added to MaxiSorp ELISA plates (Thermo Fisher, Waltham, MA, USA; #439454) coated with C.1086 UFO-v2-RQH (86). After 1 hour of blocking at room temperature (RT), diluted serum was added to the plates and allowed to incubate for 2 hours. The plates were then washed with PBST six times, and secondary antibody [anti-NHP IgG-HRP, Nordic MUbio, Netherlands #GAMon/IgG(Fc)/PO, diluted 1/10,000 in 4% whey buffer] was added, and the plates were incubated for 1 hour at RT. After another round of washing, TMB substrate and 3,3'5,5'-Tetramethylbenzidine (TMB) solution B (SeraCare, Milford, MA #5120-0047) were mixed 1:1 and added to the plates for 20 minutes at room temperature. Stopping solution (1 N H_3_PO_4_) was added, and plates were read at 450 nm. Pooled serum of known concentration was used as a standard.

Binding antibodies to a panel of HIV-1 envelope and V1V2 vaccine strain (C.1086), challenge strain (C.CH505), and globally diverse gp70 V1V2 scaffold antigens (87) were assessed in serum and rectal secretions by BAMA (9, 88, 89). Rectal secretions were eluted from Weck-Cel sponges, as previously described (90). To detect HIV-1-specific IgG or IgA, diluted serum (1:80, fivefold, six dilutions) or rectal (1:2 and 1:20 dilutions) samples were incubated with fluorescently coded antigen-coupled magnetic beads (Luminex Corporation, Austin, TX), washed, and then incubated with either goat anti-monkey IgG-biotin (Rockland, Limerick, PA) or goat anti-human IgA-biotin (Jackson Immunoresearch, West Grove, PA) (IgA measured only in rectal samples), followed by washing and incubation with PE Streptavidin (BD Pharmingen, San Diego, CA). For measuring IgA, rectal samples were first depleted of IgG by Protein G MultiTrap plate (General Electric, now Cytiva, Marlborough, MA), following the manufacturer’s protocol. Beads were acquired on a Bio-Plex^®^ 200 instrument (Bio-Rad, Hercules, CA), with antibody binding reported as mean fluorescence intensity (MFI). Assay positive controls included HIV-1 V2-specific monoclonal antibody CH58 IgG1, HIVIG (purified immunoglobulin from people living with HIV), and beads conjugated to purified rhesus IgG or IgA. Negative controls included normal human seronegative serum, blank (uncoupled; no antigen), or MuLV gp70 (empty) scaffold-conjugated beads. Positive controls were tracked with historical data using the Levey–Jennings charts to ensure consistency between assays.

For serum samples, antibody titers are reported as area under the titration curve (AUC), calculated from background (well on each plate containing beads and no sample) and blank bead or MuLV gp70 bead subtracted (net) MFI values at each dilution, using the trapezoidal method. Sera were considered positive if the net MFI at the 1:80 dilution was 1) greater than 100, 2) greater than or equal to the antigen-specific cutoff (95th percentile of all baseline sample binding per antigen after high baseline exclusion and at least 100 MFI), and 3) threefold greater than the matched baseline sample net MFI before and after negative control bead (blank or MuLV gp70 bead) subtraction.

For rectal samples, HIV-1-specific IgG or IgA was normalized to total IgG or IgA (determined by MSD Isotyping Panel 1 Human/NHP Kit, Meso Scale Discovery, Rockville, MD, LLC), calculated as SA [BAMA net MFI (for dilution in the linear range) × dilution factor divided by total antibody concentration in microgram per milliliter]. Rectal samples were considered positive if 1) the BAMA net MFI was greater than or equal to 100, 2) SA was greater than the antigen-specific cutoff (95th percentile of all baseline sample-specific activity per antigen/antibody isotype), and 3) SA was greater than threefold that of the matched baseline sample.

### Neutralization assays

Env-pseudotyped virus neutralization assays completed at Duke were measured as a function of reductions in luciferase (Luc) reporter gene expression after a single round of infection in TZM-bl cells (91, 92). TZM-bl cells (also called JC57BL-13) were obtained from the NIH AIDS Research and Reference Reagent Program, as contributed by John Kappes and Xiaoyun Wu. Briefly, a pre-titrated dose of virus was incubated with serial dilutions of heat-inactivated (56°C, 30 minutes) serum samples in duplicate for 1 hr at 37°C in 96-well flat-bottom culture plates, followed by the addition of freshly trypsinized cells. One set of control wells received cells + virus (virus control), and another set received cells only (background control). After 48–72 hours of incubation, cells were lysed and measured for luminescence using the Brit elite Luminescence Reporter Gene Assay System (PerkinElmer Life Sciences, Waltham, MA). ID_50_/IC_50_ and ID_80_/IC_80_ neutralization titers/concentrations are the dilution (serum/plasma samples) or concentration (mAbs) at which relative luminescence units (RLU) were reduced by 50% or 80% compared to virus control wells after subtraction of background RLUs from cell controls.

### Antibody-dependent cell-mediated viral inhibition

This assay was performed as previously described with some modifications (93). Briefly, on day 1, CEM NK^r^ cells were spinoculated at 1,500 ×*g* for 3 hours with SHIV.CH505.375H.dCT virus isolated from transfected 293T cells. On day 2 of the assay, cryopreserved NHP PBMCs were thawed, washed, and counted. Cells were added to a V-bottom plate (Corning Incorporated, Corning, NY, USA) at a concentration of 1 × 10^5^ cells/well and allowed to rest overnight. On day 3, serum from week 0 (baseline) and week 27 (2 weeks post-protein) was incubated for 2 hours with 1 × 10^4^ infected CEM NK^r^ cells that had been washed three times to remove unbound virus. After 2 hours of incubation, serum and infected CEM NK^r^ cells were added to PBMCs. PGT121 (catalog no. 12343; NIH AIDS Reagent Program) and EM4C04 (anti-influenza HA antibody) served as positive and negative controls, respectively. Five days after incubation, cells were washed 2×, and fresh media was added to all wells. On day 8 after incubation, plates were spun at 1,500 rpm for 5 minutes, the supernatant was harvested, and the SIV Gag p27 ELISA was performed.

Briefly, high-binding ELISA plates (Thermo Scientific, Waltham, MA, USA) were coated at 0.5 μg/mL with goat anti-mouse IgG2b (SouthernBiotech, Birmingham, AL, USA) overnight at 4 °C. The next day, plates were washed 6× with PBST and blocked with 2% goat serum in PBST for 30 minutes at RT. Anti-p27 2F12 (catalog no. 2343; NIH AIDS Reagent Program) antibody, at 0.5 μg/mL, was added to ELISA plates and incubated for 1 hour at 37 °C. Plates were again washed 6× with PBST. Immediately after harvesting, supernatants from the ADCVI assay were treated with Triton X (Sigma-Aldrich, St. Louis, MO) to make a 0.5% Triton X solution to inactivate virus particles and release Gag. Gag supernatant was diluted threefold, added to ELISA plates, and allowed to incubate for 1.5 hours at 37 °C. Plates were washed, and biotinylated SIV IG was diluted 1:1,000 and added to each well. Plates were again incubated for 1 hour at 37 °C. After 6× washes, neutralite-avidin peroxidase (N-HRP) (SouthernBiotech, Birmingham, AL, USA) was diluted 1:4,000 and added to each plate for 30 minutes at RT in the dark. After incubation, bound IgG was detected using tetramethylbenzidine substrate (KPL, Gaithersburg, MD, USA). The reaction was stopped by adding 100 μL of 2 N H_2_SO_4_.

### Antibody-dependent cellular phagocytosis

Baseline (week 0), week 27, and week 39 serum samples were tested for ADCP activity mediated by THP-1 cells (human monocytic cell line; ATCC) against C.CH505T/F_gp140 (SHIV challenge strain matched) antigen-coated beads (94). NeutrAvidin-labeled fluorescent beads (Invitrogen, Waltham, MA, USA) were conjugated to biotinylated antigen and then incubated with 1:50 diluted serum samples, positive (HIVIG), or negative control antibodies (influenza-specific mAb CH65) (tested in duplicate) to form immune complexes. THP-1 cells, pre-treated with anti-human CD4 (San Diego, CA, BioLegend), were added to immune complexes, followed by spinoculation at 4 °C and incubation at 37 °C/5% CO_2_ to allow phagocytosis to occur. Cells were fixed with paraformaldehyde and analyzed on a BD LSRFortessa flow cytometer (BD Biosciences, San Jose, CA, USA) to evaluate fluorescence. ADCP scores were calculated by multiplying the mean fluorescence intensity by the percentage of bead-positive THP-1 cells for test serum or control antibody, divided by the MFI and percentage of bead-positive cells obtained for the no-antibody (PBS) control. Post-baseline samples were considered positive when the ADCP score was three times greater than the baseline sample ADCP score and greater than the antigen/experiment-specific cutoff (95th percentile of baseline sample ADCP scores and at least 1). ADCP scores are reported from one of two independent experiments with comparable results.

### Antibody-dependent cell-mediated cytotoxicity

To evaluate the ADCC activity in serum samples, we utilized a previously established protocol incorporating EGFP-CEM-NKr-CCR5-SNAP cells, which constitutively express GFP, as target cells (40). Briefly, one million target cells were incubated with 50 μg of CH505 gp120 protein for 2 hours at 37 °C. Post-incubation, the target cells were washed and labeled with SNAP-Surface^®^ Alexa Fluor^®^ 647 (New England Biolabs, Ipswich, MA, USA) according to the manufacturer’s instructions for 30 minutes at room temperature. Heat-inactivated serum samples (56 °C for 30 minutes) were serially diluted (seven 10-fold dilutions starting at 1:10), and 100 μL of each dilution was added to the wells of a 96-well V-bottom plate (Millipore Sigma, Burlington, MA, USA). To each well, 50 μL containing 5,000 target cells and 50 μL containing 250,000 human PBMCs were added, achieving an effector-to-target (E/T) ratio of 50:1. The plate was incubated for 2 hours at 37 °C, followed by two washes with PBS. The cells were then resuspended in 200 μL of a 1% PBS–paraformaldehyde solution and analyzed using a Symphony flow cytometer equipped with a high-throughput system (BD Biosciences, San Jose, CA, USA). Specific cytotoxicity was determined by the loss of GFP from the SNAP-Alexa647^+^ target cells. Background levels were established using target and effector cells cultured in R10 media alone.

To evaluate V2-specific ADCC, F(ab’)2 fragments were generated from CAP228-16H and CAP228-3D monoclonal antibodies using the Pierce F(ab’)2 Micro Preparation Kit (cat. #44688, Thermo Fisher, Waltham, MA, USA) in accordance with the manufacturer’s protocol. To confirm the purity of the recovered F(ab’)2 fragments, sodium dodecyl sulfate-polyacrylamide gel electrophoresis (SDS–PAGE) was performed, followed by silver staining using the Silver Quest Staining Kit (cat. #LC6070, Invitrogen, Waltham, MA, USA), again following the provided instructions. The V2-specific ADCC activity was subsequently assessed (11, 20, 21). Target cells, coated with CH505 gp120 protein and subsequently labeled with SNAP-Surface^®^ Alexa Fluor^®^ 647, were incubated with 5 μg/mL of the purified F(ab’)2 fragments from CAP228-16H or CAP228-3D monoclonal antibodies for 1 hour at 37 °C. These prepared target cells were then subjected to ADCC assay as outlined above. Target cells incubated without F(ab’)2 fragments were used as the total ADCC response. The presence of F(ab’)2 fragments inhibits the binding and ADCC mediated by anti-V2 antibodies present in the plasma of immunized animals. The specific V2-specific ADCC activity was determined by subtracting the percentage of ADCC activity in the presence and absence of the F(ab’)2 fragments.

### IgG subclass

To measure IgG subclass, 10 rows of an Immulon 4 HXB 96 well plate (VWR, Radnor, PA, USA) were coated overnight at 4°C with 50 ng per well of consensus C cyc P gp120 or biotinylated CH505 Env protein in PBS at pH 7.2. The remaining two rows of the plate were coated with 50 ng per well of recombinant human CD40 (NHP Reagent Resource) to generate a standard curve. The following day, the plate was washed with PBS containing 0.05% Tween-20 and blocked with 0.1% bovine serum albumin (BSA) in PBST. After 15 minutes, the block buffer was removed. Duplicate twofold serial dilutions containing 1.5–100 ng/mL of recombinant anti-CD40 rhesus IgG1, -IgG2, -IgG3, or -IgG4 monoclonal antibody (NHP Reagent Resource) in 0.1% BSA/PBST were placed in the CD40-coated wells. Serial dilutions of test serum were added to wells coated with Env protein. Following overnight storage at 4°C, the plate was washed and incubated with the appropriate monoclonal antibody (from the NHP Reagent Resource): anti-rhesus IgG1 (clone 3.10C.3; mouse IgG2c), -IgG2 (clone dio; mouse IgG1), -IgG3 (clone tria; mouse IgG1), or -IgG4 (clone tessara; mouse IgG1). After 1 hour at 37°C, the plate was washed and reacted for 30 minutes at 37°C with horseradish peroxidase (HRP)-labeled goat anti-mouse IgG2c or -IgG1 (both from SouthernBiotech, Birmingham, AL, USA). After washing, 100 µL tetramethylbenzidine substrate (Surmodics, Eden Prairie, MN, USA) was added to each well. Absorbance at 370 nm was recorded 30 minutes later in a SpectraMax M5 (Molecular Devices, San Jose, CA, USA). Concentrations of anti-Env IgG subclass antibody were subsequently interpolated from a five-parameter standard curve constructed using the absorbance values and concentrations of anti-CD40 monoclonal antibody in the CD40-coated wells.

### Flow cytometry-based phenotypic characterization and intracellular cytokine staining assay

Antigen-specific cytokine production in PBMCs and rectal biopsies was measured via ICS. PBMCs and rectal biopsies were processed as previously described (28). Cells were then stimulated at 37°C and 5% CO_2_ with anti-CD28 (BD Pharmingen, #555725), anti-CD49d (BD Pharmingen, #555501), and 1 μg/mL of either Gag, Env, or ConC-CycP gp120 peptide pools. phorbol myristate acetate (PMA)/ionomycin was used as a positive control. After 2 hours, Golgi Stop and Golgi Plug (BD Biosciences, #554724 and #555029) were added to the cells and allowed to incubate for another 4 hours. The stimulated cells were then placed at 4°C overnight before staining. The next morning, the PBMCs were washed in fluorescence-activated cell sorting (FACS) buffer, and a cocktail of surface marker-specific antibodies was added (full panel can be found in **Supplementary Tables S1**, **S2**). After a 30-minute incubation at 4°C, cells were washed in FACS buffer and fixed using Cytofix/Cytoperm (BD Biosciences, #554722) for 20 minutes at 4°C. Cells were washed twice in perm wash buffer (BD Biosciences, #554723), and a cocktail of cytokine-specific antibodies was added for a 30-minute stain at 4°C (**Supplementary Tables S1**, **S2**). Cells were washed twice again, first in perm wash and then in FACS buffer, and then acquired on an LSR II Flow Cytometer.

Blood T-cell phenotype and innate cell phenotype were measured via flow cytometry on thawed PBMCs. Briefly, frozen PBMCs from weeks 0, 1, 2, and 9 post-protein were thawed and stained for T-cell phenotype, and PBMCs from days 0, 1, and 2 were thawed and stained for innate phenotype. For cells being stained for T-cell phenotype, live/dead stain Zombie (BioLegend, #423113) was diluted in PBS and added. After a 30-minute RT incubation, cells were washed in FACS buffer. Cocktails of surface marker-specific antibodies were then added to cells being stained for innate phenotype as well as T-cell phenotype (full panels can be found in **Supplementary Tables S3**, **S4**). The innate phenotype panel contained a Near-IR live/dead stain instead of Zombie. After another 30-minute incubation, FACS lysing solution (BD Biosciences, #349202) was added and allowed to incubate for 10 minutes at RT. Permeabilization buffer diluted to 1× (Fisher, #50-112-9082, #50-112-908) was then added to the cells and allowed to incubate for 30 minutes at 4°C. Cells were washed twice in 1× perm wash buffer (Fisher, #50-112-9059), and cells were intracellularly stained for Ki67 (BD Biosciences, #561277). After another 30-minute incubation at RT, cells were washed once more in perm wash buffer and then in FACS wash. Cells were acquired on an LSR II Flow Cytometer.

Rectal biopsy T-cell phenotype was measured on fresh cells after being processed as previously described (28). All steps were the same as above except that the live/dead stain (Fisher Scientific, #L34976) was included in the surface marker stain (full panel can be found in **Supplementary Table S5**).

### Innate cytokine analysis using Meso Scale Discovery

The MSD U-PLEX Viral Combo 1 (NHP) kit (cat. # K15344K-1) was used to measure cytokine concentration in plasma and cell supernatant. The kit measures 19 analytes: G-CSF, GM-CSF, IFN-α2a, IFNγ, IL-1RA, IL-1β, IL-4, IL-5, IL-6, IL-7, IL-8, IL-9, IL-10, IL-12p70, IP-10, MCP-1, MIP-1α, TNF-α, and VEGF-A. The MSD-provided protocol for U-PLEX assays was followed with no changes. Briefly, after bringing all reagents to room temperature, the two multiplex coating solutions were added to the appropriate 96-well plate (50 μL per well), and the plates were covered and incubated for 1 hour while shaking at 600 rpm on a Lab-Line Instruments Titer plate shaker. After washing three times with 1× MSD wash buffer (cat. # R61AA-1), two replicates of serial fivefold dilutions of calibrator mixes were added to the appropriate plates, along with two blank wells. Plasma was diluted twofold and added to the appropriate wells (50 μL per well). Plates were incubated while shaking for 1 hour and washed again, and then the detection antibody master mix was added to the appropriate plates. After another 1-hour incubation and another wash, MSD GOLD Read Buffer B was added to each well, and the plate was immediately read using the MESO QuickPlex SQ 120 MM instrument.

### Statistical analysis

All statistical analyses were performed using the GraphPad Prism v 10 software. The threshold for statistical significance (alpha) is 0.05, and trends are defined as *p* < 0.06 for all statistical tests. The Kaplan–Meier curves and log-rank Mantel–Cox test were used to determine differences in the rate of acquisition of infection between the vaccinated and unvaccinated control groups. For datasets where both changes over time and differences between groups were of interest, repeated-measures two-way ANOVA was used when conditions were met, unless values were missing, in which case mixed-effects analysis was used. Follow-up tests to both repeated-measures two-way ANOVA and mixed-effects analyses were performed using Dunnett’s multiple comparison test for comparing baseline to each subsequent timepoint, and Fisher’s LSD between groups at each timepoint. When conditions for two-way ANOVA were not met, non-parametric tests were used. Namely, the Kruskal–Wallis with Dunn’s multiple comparison follow-up was used to compare changes over time, and the Mann–Whitney tests were used to compare groups at each timepoint. For comparisons of binding between different antigens, Wilcoxon tests were used. For comparisons of more than two groups at the same timepoint, after checking that conditions were satisfied, one-way ANOVA was used with Tukey’s multiple comparison follow-up. When conditions were satisfied, unpaired t-tests were used for comparisons of two groups at a single timepoint, and Welch’s correction was used when the equal variance assumption was not met. For comparisons of two groups of non-parametric data, the Mann–Whitney tests were used. Spearman’s correlation coefficients were calculated for measuring correlations, and two-sided *p*-values are indicated. Adjusted *p*-values are reported when multiple comparison correction was used. *p < 0.05, **p < 0.01, ***p < 0.001, and ****p < 0.0001.

## Data Availability

The original contributions presented in the study are included in the article/**Supplementary Material**. Further inquiries can be directed to the corresponding author.

## Author disclaimer

This research was supported in part by the Intramural Research Program of the National Institutes of Health (NIH). The contributions of the NIH authors were made as part of their official duties as NIH federal employees, are in compliance with agency policy requirements, and are considered Works of the United States Government. However, the findings and conclusions presented in this paper are those of the authors and do not necessarily reflect the views of the NIH or the U.S. Department of Health and Human Services.

## Supplementary material

Supplementary Figure 1 Individual viral load kinetics. **(A)** Viral load of unvaccinated control (n = 8 infected), alum (n = 9 infected), and dmLT (n = 6 infected) groups from weeks 0–12 post-infection shown by individual animal. Gray background indicates threshold for positive viral load (n = 60 copies/mL).

Supplementary Figure 2 Supplemental rectal secretion antibody data. **(A)** Rectal secretion IgA specific activity (SA, MFI x dilution/Total IgG μg/mL) for **(C)** 1086 (left) and CH505 (right) gp140 at week 0, 27, and 39 of the study. Gray background indicates antigen-specific threshold. **(D)** Spearman correlation of Serum binding antibody and rectal secretion IgG SA for SHIV CH505 gp140 at week 2 post-protein.

Supplementary Figure 3 Supplemental antibody function data. **(A)** Neutralization of SHIV.C.CH505.375H (challenge strain) and Ce1086_B2 Env-pseudotyped viruses at week 0, 27, and 39 of the study. Both virus strains are Tier 2. **(B)** V2-specific antibody-dependent cellular cytotoxicity (ADCC) at week 0 and 27 of the study determined by subtracting ADCC activity with CAP228-3D FAb (left) and CAP228-16H FAb (right) from total ADCC. Two-Way ANOVA with Fisher’s LSD test follow up. All panels: Gray background indicates threshold of detection. Box indicates 25^th^–75^th^ percentile, horizontal bar indicates median, whiskers indicate min–max.

Supplementary Figure 4 Supplemental T cell response data. **(A)** IFNγ^+^ as a percent of CD4 T cells in PBMCs after stimulation with Env (ICS) at MVA timepoints (week 0, 2, and 11 of the study). **(B)** IFNγ^+^ as a percent of CD8 T cells in PBMCs after stimulation with Env (ICS) at week 0, 2, 11, 26, and 39 of the study. **(C, D)** IFNγ^+^ as a percent of **(C)** CD4 T cells and **(D)** CD8 T cells in rectal biopsies after stimulation with Env (ICS) at week 0, 2, 11, 26, 35, and 39 of the study. **(E)** Representative flow plots of (top to bottom) Ki67^+^ on CD4 T_CM_, α4β7^+^ on Ki67^+^ CD4 T_CM_, and ICOS^+^Ki67^+^ on CD4 T cells at week 0 and 1 post-protein. **(F)** Ki67^+^ as a percent of CD4 T cells at week 0, 1, 2, and 9 post-protein. **(G)** Frequency week 1 post-protein in early infected (≤ 5 challenges to infection) vs late and uninfected (> 5 challenges to infection) animals. **(H)** α4β7^+^ as a percent of Ki67^+^ CD4 T cells in PBMCs at week 0, 1, 2, and 9 post-protein. **(I)** α4β7 frequency on Env-specific, IFNγ^+^ CD4 T cells at week 1 post-protein. **(J)** (left) Ki67^+^ CXCR3 single-positive cells and (right) Ki67^+^ CCR4 single-positive on CD4 T cells at week 0 and 1 post-protein. “X3” is CXCR3, “R4” is CCR4, and “R6” is CCR6. Statistics: **(A, B, F, H)**: Two-way ANOVA, or (**C, D**, which contain missing values at some timepoints) Mixed-effects analysis (Follow up: Dunnett’s multiple comparisons test between baseline and later timepoints, and Fisher’s LSD between groups). **(J)** Two-way ANOVA (Follow up: Fisher’s LSD). **(G, I)** Unpaired T test, with Welch’s correction for **(I)**. All panels: gray stars indicate significant difference from baseline, black stars with brackets indicate significant difference between groups. **(A–D)** Gray background indicates threshold for positive response. **(A–D, F–J)** Box indicates 25^th^–75^th^ percentile, horizontal bar indicates median, whiskers indicate min–max. * *p* < 0.05, ** *p* < 0.01, *** *p* < 0.001.

Supplementary Figure 5 Supplemental rectal biopsy T cell data. **(A–F)** In order, α4β7^+^Ki67^+^, α4β7^+^CCR5^+^Ki67^+^, T_RM_ (CD69^+^CD103^+^), α4β7^+^ T_RM_, α4β7^+^CCR5^+^ T_RM_, and CCR5^+^ T_RM_ as a percent of CD4 T cells in rectal biopsies at 4 weeks pre-challenge. **(G, H)** Ki67^+^**(G)** and T_RM_ (CD69^+^CD103^+^) **(H)** as a percent of CD8 T cells in rectal biopsies at 4 weeks pre-challenge. Statistics for all: One-way ANOVA (Follow up: Tukey’s multiple comparison). Box indicates 25^th^–75^th^ percentile, horizontal bar indicates median, whiskers indicate min–max. All panels: gray stars indicate significant difference from baseline, black stars with brackets indicate significant difference between groups.

Supplementary Figure 6 Supplemental innate cell data. **(A, D, E)** Mean frequency of CD80^+^ and CD86^+^ as a percent of **(A)** classical monocytes, **(D)** pDCs, and **(E)** CD11c+BDCA1- DCs at day 0, 1 and 2 post-protein. **(B, C, F–H)** Mean frequency of CD80^+^, CD86^+^, and CD80^-^CD86^-^ as a percent of **(B)** intermediate and **(C)** non-classical monocytes, **(F)** CD11c^+^BDCA1^+^ DCs, **(G)** CD11c^-^BDCA1^-^ DCs, and **(H)** CD11c^-^BDCA1^+^ DCs at day 0, 1, and 2 post-protein. **(I)** Mean plasma concentration of (left to right) MCP-1, MIP-1α, G-CSF, IL-7, IL-8, IL-9, IL-10, IL-1RA and VEGF-A at day 0, 1, and 2 post-protein. Statistics: Two-way ANOVA (Follow up: Dunnett’s multiple comparisons test between baseline and later timepoints, and Fisher’s LSD between groups at each timepoint). Error bars indicate SEM. All panels: orange and blue stars indicate significant difference from baseline within alum or dmLT (respectively), black stars indicate significant difference between groups. * *p* < 0.05, ** *p* < 0.01.

Supplementary Figure 7 Infection curves with Mamu-A*01^+^ animals. **(A)** Acquisition of SHIV.CH505 through intrarectal challenge of unvaccinated control animals triple negative for Mamu-A*01, Mamu-B*08, and Mamu-B*17 (n = 9, Controls Triple -, green) and animals positive for one or more of these alleles (n = 6, Controls Allele +, purple). Five of these animals (1 triple – and 4 allele +) were challenged in a separate study. **(B)** Acquisition of SHIV.CH505 through intrarectal challenge of unvaccinated control, alum, and dmLT groups with Mamu-A*01 animals included (n = 10 for all groups). Statistics: Kaplan-Meier infection curves, Log-rank Mantel-Cox test.

Supplementary Table 1 ICS flow cytometry antibody panel for PBMCs.

Supplementary Table 2 ICS flow cytometry antibody panel for rectal biopsy.

Supplementary Table 3 T cell phenotype flow cytometry antibody panel for PBMCs.

Supplementary Table 4 Innate phenotype flow cytometry antibody panel for PBMCs.

Supplementary Table 5 T cell phenotype flow cytometry antibody panel for rectal biopsy.
