# Supplementary figures and images for: Immunogenicity and protection mediated by dmLT and alum adjuvants for an HIV-1 vaccine
Source: Front Immunol. 2026 Jan 21;16:1706958. doi: 10.3389/fimmu.2025.1706958 (PMC12867785; doi:10.3389/fimmu.2025.1706958)

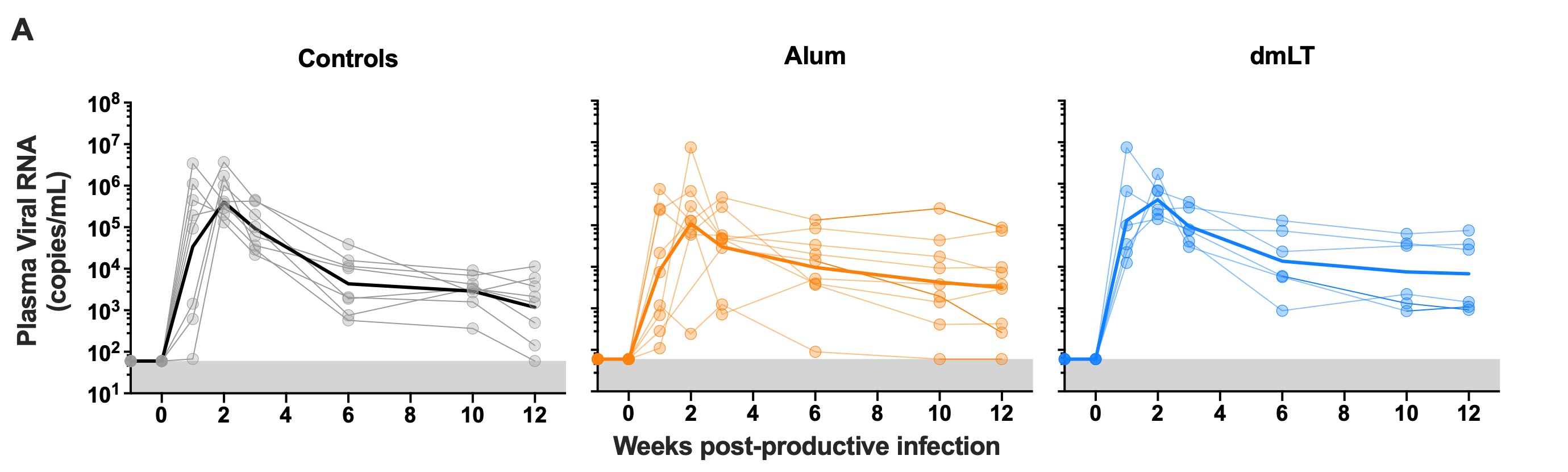

Supplement: Supplementary Figure 1 — Individual viral load kinetics. (A) Viral load of unvaccinated control (n = 8 infected), alum (n = 9 infected), and dmLT (n = 6 infected) groups from weeks 0–12 post-infection shown by individual animal. Gray background indicates threshold for positive viral load (n = 60 copies/mL). [file Image1.jpeg]

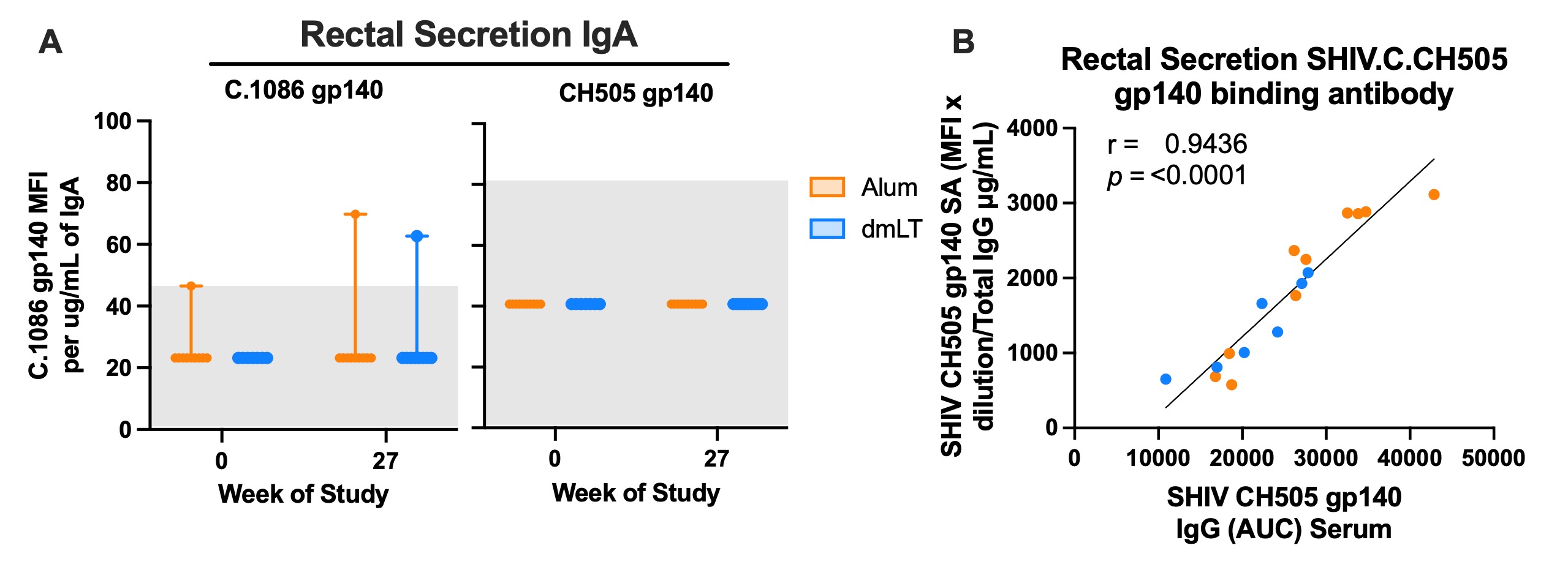

Supplement: Supplementary Figure 2 — Supplemental rectal secretion antibody data. (A) Rectal secretion IgA specific activity (SA, MFI x dilution/Total IgG μg/mL) for (C) 1086 (left) and CH505 (right) gp140 at week 0, 27, and 39 of the study. Gray background indicates antigen-specific threshold. (D) Spearman correlation of Serum binding antibody and rectal secretion IgG SA for SHIV CH505 gp140 at week 2 post-protein. [file Image2.jpeg]

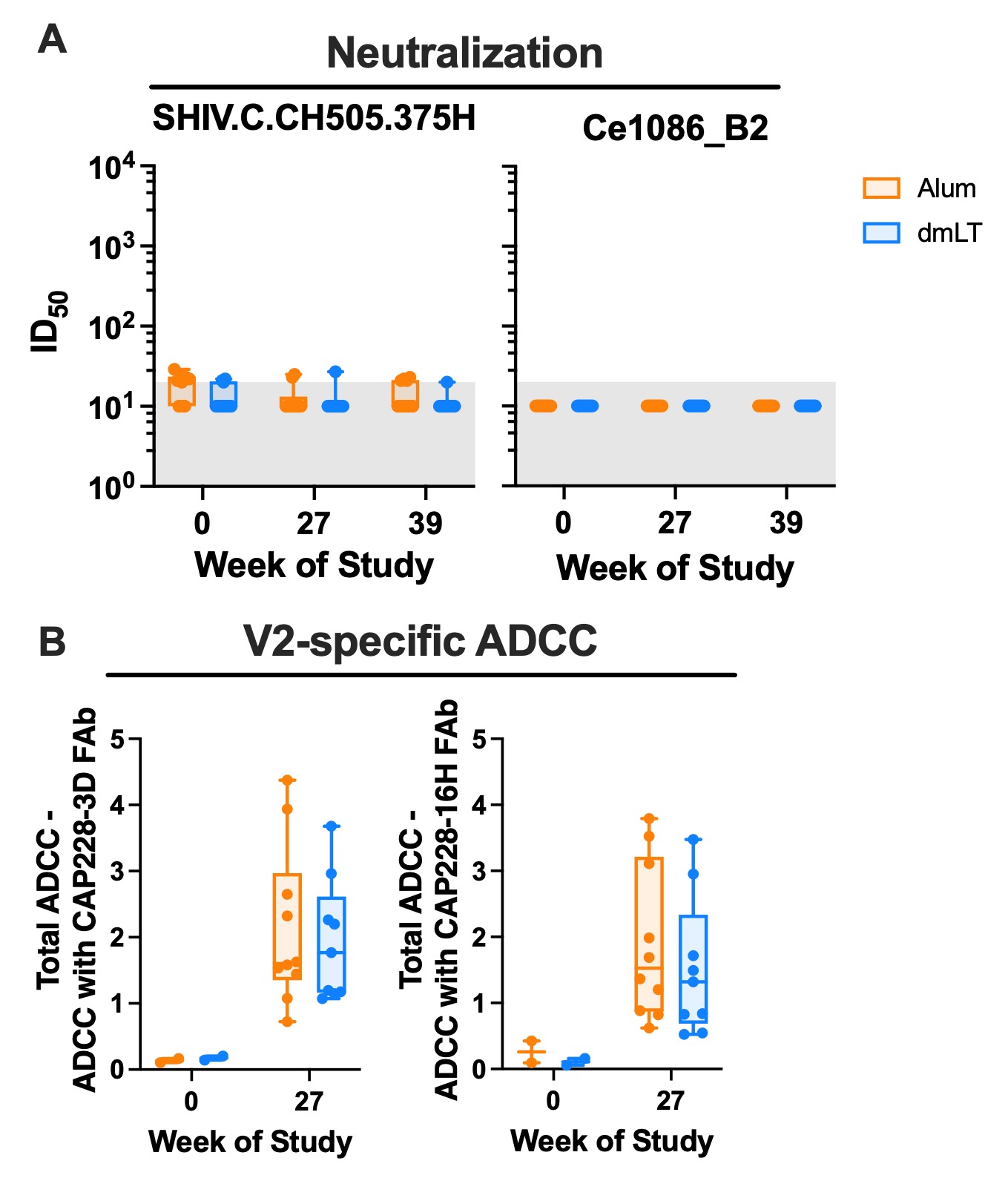

Supplement: Supplementary Figure 3 — Supplemental antibody function data. (A) Neutralization of SHIV.C.CH505.375H (challenge strain) and Ce1086_B2 Env-pseudotyped viruses at week 0, 27, and 39 of the study. Both virus strains are Tier 2. (B) V2-specific antibody-dependent cellular cytotoxicity (ADCC) at week 0 and 27 of the study determined by subtracting ADCC activity with CAP228-3D FAb (left) and CAP228-16H FAb (right) from total ADCC. Two-Way ANOVA with Fisher’s LSD test follow up. All panels: Gray background indicates threshold of detection. Box indicates 25th–75th percentile, horizontal bar indicates median, whiskers indicate min–max. [file Image3.jpeg]

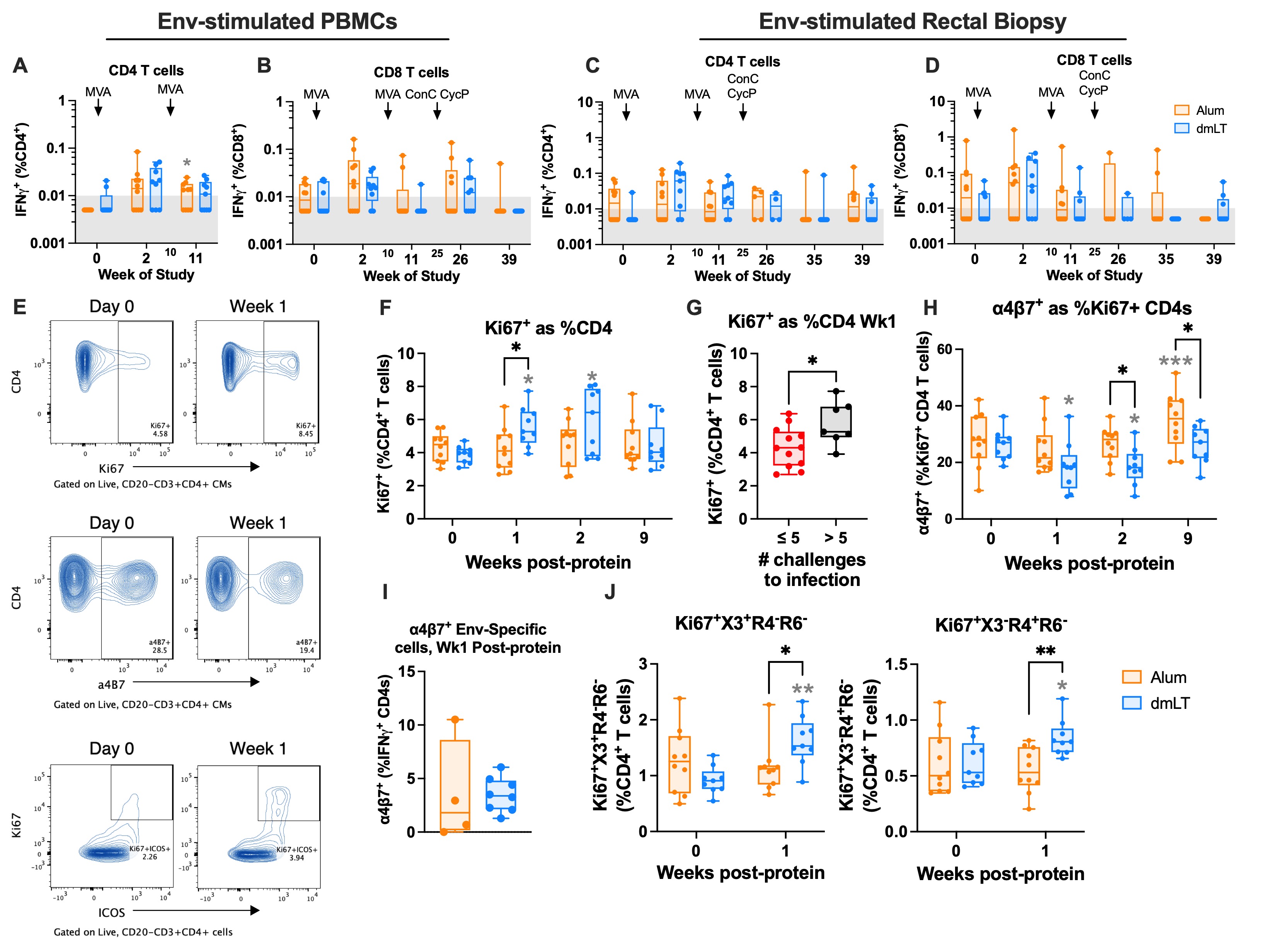

Supplement: Supplementary Figure 4 — Supplemental T cell response data. (A) IFNγ+ as a percent of CD4 T cells in PBMCs after stimulation with Env (ICS) at MVA timepoints (week 0, 2, and 11 of the study). (B) IFNγ+ as a percent of CD8 T cells in PBMCs after stimulation with Env (ICS) at week 0, 2, 11, 26, and 39 of the study. (C, D) IFNγ+ as a percent of (C) CD4 T cells and (D) CD8 T cells in rectal biopsies after stimulation with Env (ICS) at week 0, 2, 11, 26, 35, and 39 of the study. (E) Representative flow plots of (top to bottom) Ki67+ on CD4 TCM, α4β7+ on Ki67+ CD4 TCM, and ICOS+Ki67+ on CD4 T cells at week 0 and 1 post-protein. (F) Ki67+ as a percent of CD4 T cells at week 0, 1, 2, and 9 post-protein. (G) Frequency week 1 post-protein in early infected (≤ 5 challenges to infection) vs late and uninfected (> 5 challenges to infection) animals. (H) α4β7+ as a percent of Ki67+ CD4 T cells in PBMCs at week 0, 1, 2, and 9 post-protein. (I) α4β7 frequency on Env-specific, IFNγ+ CD4 T cells at week 1 post-protein. (J) (left) Ki67+ CXCR3 single-positive cells and (right) Ki67+ CCR4 single-positive on CD4 T cells at week 0 and 1 post-protein. “X3” is CXCR3, “R4” is CCR4, and “R6” is CCR6. Statistics: (A, B, F, H): Two-way ANOVA, or (C, D, which contain missing values at some timepoints) Mixed-effects analysis (Follow up: Dunnett’s multiple comparisons test between baseline and later timepoints, and Fisher’s LSD between groups). (J) Two-way ANOVA (Follow up: Fisher’s LSD). (G, I) Unpaired T test, with Welch’s correction for (I). All panels: gray stars indicate significant difference from baseline, black stars with brackets indicate significant difference between groups. (A–D) Gray background indicates threshold for positive response. (A–D, F–J) Box indicates 25th–75th percentile, horizontal bar indicates median, whiskers indicate min–max. * p < 0.05, ** p < 0.01, *** p < 0.001. [file Image4.jpeg]

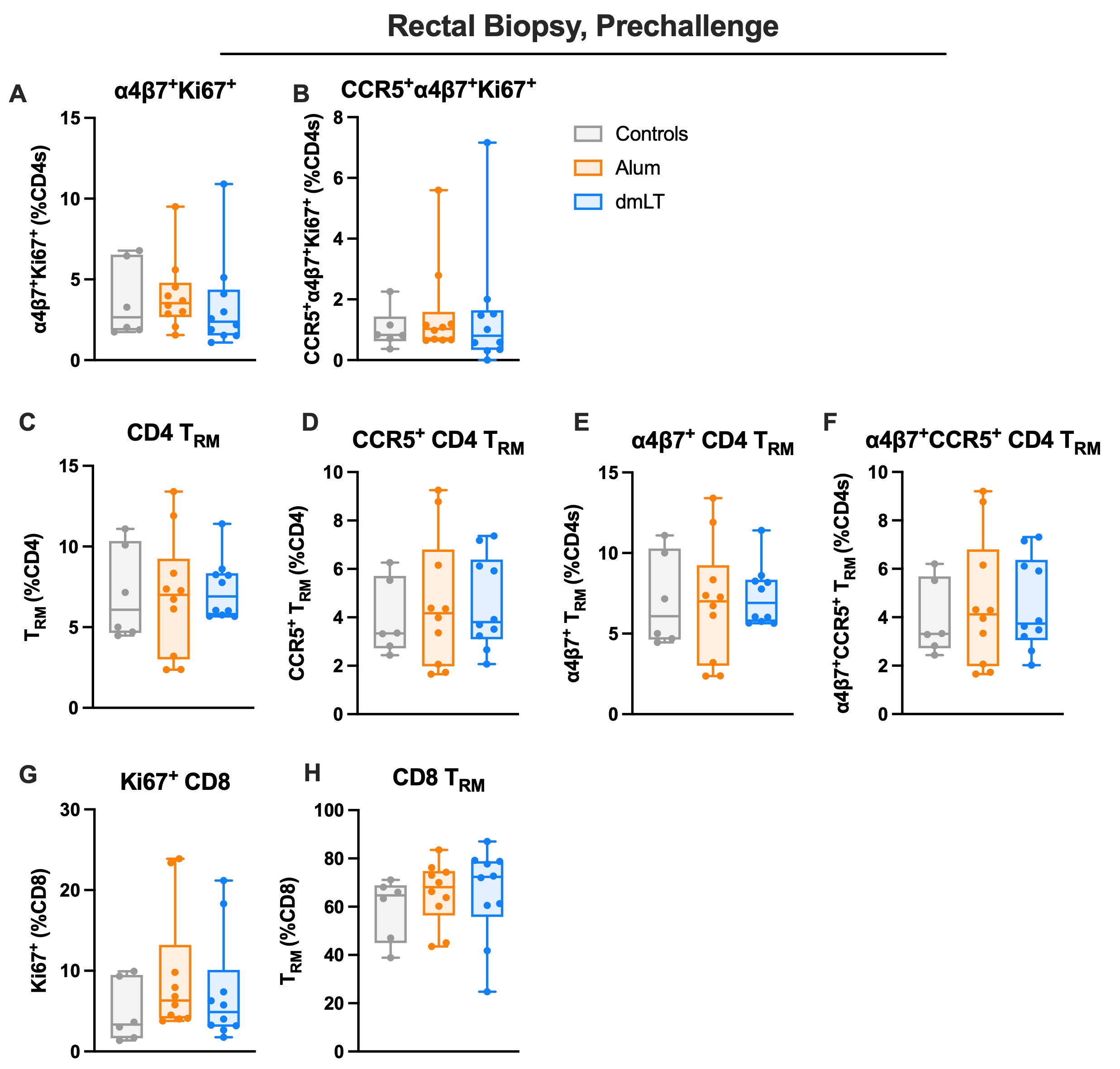

Supplement: Supplementary Figure 5 — Supplemental rectal biopsy T cell data. (A–F) In order, α4β7+Ki67+, α4β7+CCR5+Ki67+, TRM (CD69+CD103+), α4β7+ TRM, α4β7+CCR5+ TRM, and CCR5+ TRM as a percent of CD4 T cells in rectal biopsies at 4 weeks pre-challenge. (G, H) Ki67+(G) and TRM (CD69+CD103+) (H) as a percent of CD8 T cells in rectal biopsies at 4 weeks pre-challenge. Statistics for all: One-way ANOVA (Follow up: Tukey’s multiple comparison). Box indicates 25th–75th percentile, horizontal bar indicates median, whiskers indicate min–max. All panels: gray stars indicate significant difference from baseline, black stars with brackets indicate significant difference between groups. [file Image5.jpeg]

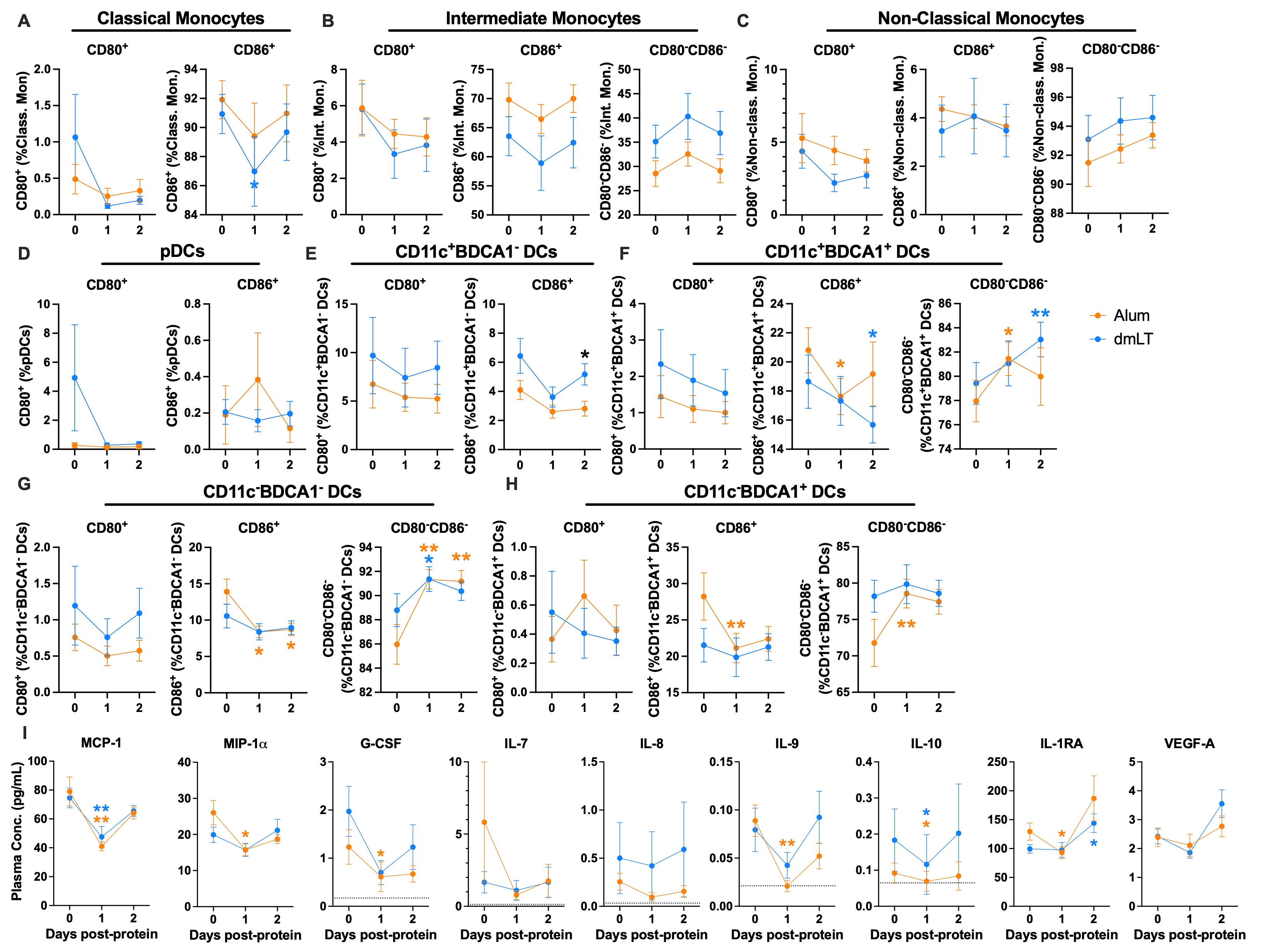

Supplement: Supplementary Figure 6 — Supplemental innate cell data. (A, D, E) Mean frequency of CD80+ and CD86+ as a percent of (A) classical monocytes, (D) pDCs, and (E) CD11c+BDCA1- DCs at day 0, 1 and 2 post-protein. (B, C, F–H) Mean frequency of CD80+, CD86+, and CD80-CD86- as a percent of (B) intermediate and (C) non-classical monocytes, (F) CD11c+BDCA1+ DCs, (G) CD11c-BDCA1- DCs, and (H) CD11c-BDCA1+ DCs at day 0, 1, and 2 post-protein. (I) Mean plasma concentration of (left to right) MCP-1, MIP-1α, G-CSF, IL-7, IL-8, IL-9, IL-10, IL-1RA and VEGF-A at day 0, 1, and 2 post-protein. Statistics: Two-way ANOVA (Follow up: Dunnett’s multiple comparisons test between baseline and later timepoints, and Fisher’s LSD between groups at each timepoint). Error bars indicate SEM. All panels: orange and blue stars indicate significant difference from baseline within alum or dmLT (respectively), black stars indicate significant difference between groups. * p < 0.05, ** p < 0.01. [file Image6.jpg]

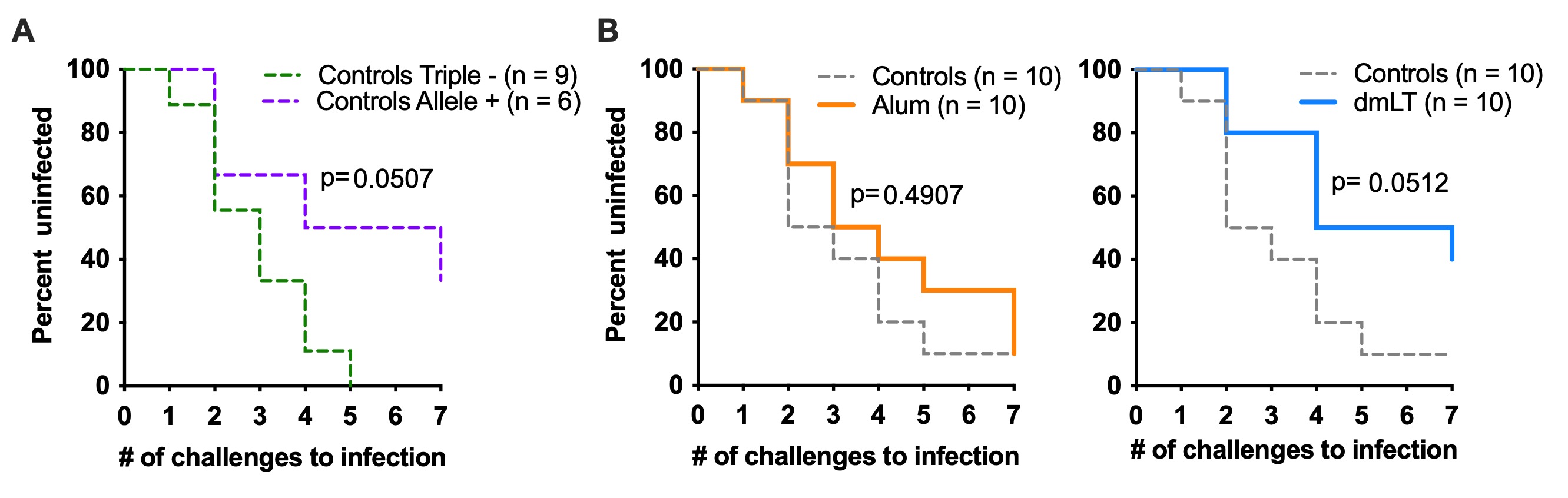

Supplement: Supplementary Figure 7 — Infection curves with Mamu-A*01+ animals. (A) Acquisition of SHIV.CH505 through intrarectal challenge of unvaccinated control animals triple negative for Mamu-A*01, Mamu-B*08, and Mamu-B*17 (n = 9, Controls Triple -, green) and animals positive for one or more of these alleles (n = 6, Controls Allele +, purple). Five of these animals (1 triple – and 4 allele +) were challenged in a separate study. (B) Acquisition of SHIV.CH505 through intrarectal challenge of unvaccinated control, alum, and dmLT groups with Mamu-A*01 animals included (n = 10 for all groups). Statistics: Kaplan-Meier infection curves, Log-rank Mantel-Cox test. [file Image7.jpeg]
