# Supplementary material for: Immunogenicity and protection mediated by dmLT and alum adjuvants for an HIV-1 vaccine
Source: Front Immunol. 2026 Jan 21;16:1706958. doi: 10.3389/fimmu.2025.1706958 (PMC12867785; doi:10.3389/fimmu.2025.1706958)
Supplement: Supplementary Table 1 — ICS flow cytometry antibody panel for PBMCs. [file Supplementaryfile1.docx]

| **PBMCs ICS panel** | | | | |
| --- | --- | --- | --- | --- |
| **Surface Markers** | **Flourophore** | **Clone** | **Company** | **Cat #** |
| A4B7 | APC | A4B7R1 | NHPRR | AB_2819257 |
| Live/Dead | Near-IR (780) | - | Thermofisher | L34976 |
| CD4 | BV650 | L200 | BD Biosciences | 563737 |
| CCR5 (CD195) | BV786 | 3A9 | BD Biosciences | 565001 |
| CD3 | PerCP-Cy5.5 | SP34-2 | BD Biosciences | 552852 |
| CD8α | BUV496 | RPA-T8 | BD Biosciences | 612942 |
| **Intracellular Markers** | **Flourophore** | **Clone** | **Company** | **Cat #** |
| IFNγ | A700 | B27 | BD Biosciences | 561024 |
| IL-2 | BV605 | MQ1-17H12 | Biolegend | 500332 |
| IL-17A | FITC | eBio64DEC17 | Thermofisher | 11-7179-42 |
| IL-4 | PE | 7A3-3 | Miltenyi | 130-123-698 |
| TNFα | PE-CF594 | MAb11 | BD Biosciences | 562784 |

**Table S1:** ICS flow cytometry antibody panel for PBMCs.

| **Rectal Biopsy ICS panel** | | | | |
| --- | --- | --- | --- | --- |
| **Surface Markers** | **Flourophore** |  | **Company** | **Cat #** |
| A4B7 | APC | A4B7R1 | NHPRR | AB_2819257 |
| Live/Dead | Near-IR | - | Thermofisher | L34976 |
| NKp44 (CD336) | BV421 | p44-8 | BD Biosciences | 744299 |
| CD4 | BV650 | L200 | BD Biosciences | 563737 |
| CCR5 (CD195) | BV786 | 3A9 | BD Biosciences | 565001 |
| CD3 | PerCP-Cy5.5 | SP34-2 | BD Biosciences | 552852 |
| NKG2A | PE-Vio770 | REA110, Z199 | Miltenyi Biotech | 130-113-567 |
| CD45 | BUV395 | D058-1283 | BD Biosciences | 564099 |
| CD8α | BUV496 | RPA-T8 | BD Biosciences | 612942 |
| **Intracellular Markers** | **Flourophore** | **Clone** | **Company** | **Cat #** |
| IFNγ | A700 | B27 | BD Biosciences | 561024 |
| IL-2 | BV605 | MQ1-17H12 | Biolegend | 500332 |
| IL-17A | FITC | eBio64DEC17 | Thermofisher | 11-7179-42 |
| IL-4 | PE | 7A3-3 | Miltenyi | 130-123-698 |
| TNFα | PE-CF594 | MAb11 | BD Biosciences | 562784 |

**Table S2:** ICS flow cytometry antibody panel for rectal biopsy.

| **Frozen PBMCs T cell phenotype panel** | | | | |
| --- | --- | --- | --- | --- |
| **Surface Markers** | **Fluorophore** | **Clone** | **Company** | **Cat #** |
| Live/Dead | Zombie Violet | - | Biolegend | 423113 |
| NKG2A (CD159a) | APC | REA110, Z199 | Milltenyi | 130-113-563 |
| ICOS (CD278) | APC-Cy7 | C398.4A | Biolegend | 313530 |
| CCR6 | BV510 | 11A9 | BD Biosciences | 563241 |
| PD-1 | BV605 | EH12.2H7 | Biolegend | 329924 |
| CD4 | BV650 | L200 | BD Biosciences | 563737 |
| CD95 | BV711 | DX2 | BD Biosciences | 563132 |
| CCR5 (CD195) | BV786 | 3A9 | BD Biosciences | 565001 |
| CXCR3 | FITC | G025H7 | Biolegend | 353704 |
| CD3 | BB700 | SP34-2 | BD Biosciences | 566517 |
| a4B7 | PE | A4B7R1 | NHPRR | AB_2819258 |
| CCR4 (CD194) | PE-CF594 | 1G1 | BD Biosciences | 565391 |
| CXCR5 (CD185) | PE-Cy7 | MU5UBEE | Thermofisher | 25-9185-42 |
| CD20 | BUV395 | 2H7 | BD Biosciences | 563782 |
| CD8 | BUV496 | RPA-T8 | BD Biosciences | 612942 |
| CD28 | BUV737 | CD28.2 | BD Biosciences | 612815 |
| **Intracellular marker** | **Fluorophore** | **Clone** | **Company** | **Cat #** |
| Ki67 | AF700 | B56 | BD Biosciences | 561277 |

**Table S3:** T cell phenotype flow cytometry antibody panel for PBMCs.

| **Frozen PBMCs Innate cell phenotype panel** | | | | |
| --- | --- | --- | --- | --- |
| **Surface Markers** | **Flourophore** | **Clone** | **Company** | **Cat #** |
| CD66abce | APC | TET2 | Miltenyi Biotech | 130-118-539 |
| Live/Dead | Near-IR (780) | - | Thermofisher | L34976 |
| CD14 | BV510 | M5E2 | Biolegend | 301842 |
| CD86 | BV605 | IT2.2 | Biolegend | 305430 |
| CD11c | BV650 | SHCL-3 | BD Bioscience | 744437 |
| BDCA1, CD1c | BV711 | L161 | Biolegend | 331536 |
| CD123 | BV786 | 7G3 | BD Biosciences | 564196 |
| BDCA3, CD141 | BB515 | 1A4 | BD Biosciences | 565084 |
| HLA-DR | BB700 | G46-6 | BD Biosciences | 566480 |
| CD80 | PE | L307.4 | BD Biosciences | 557227 |
| NKG2A | PE-Vio770 | REA110, Z199 | Miltenyi Biotech | 130-113-567 |
| CD16 | BUV395 | 3G8 | BD Biosciences | 563785 |
| CD20 | BUV496 | 2H7 | BD Biosciences | 749954 |
| CD3 | BUV737 | SP34-2 | BD Biosciences | 568353 |
| **Intracellular marker** | **Fluorophore** | **Clone** | **Company** | **Cat #** |
| Ki67 | AF700 | B56 | BD Biosciences | 561277 |

**Table S4:** Innate phenotype flow cytometry antibody panel for PBMCs.

| **Rectal Biopsy T cell phenotype panel** | | | | |
| --- | --- | --- | --- | --- |
| **Surface Markers** | **Fluorophore** | **Clone** | **Company** | **Cat #** |
| CD103 | APC | 2G5 | Beckman | B06204 |
| Live/Dead | Near-IR (780) | - | Thermofisher | L34976 |
| MR1 (TCR Va7.2) | BV421 | 3C10 | Biolegend | 351716 |
| CCR6 | BV510 | 11A9 | BD Biosciences | 563241 |
| CD20 | BV605 | 2H7 | Biolegend | 302334 |
| CD4 | BV650 | L200 | BD Biosciences | 563737 |
| CD95 | BV711 | DX2 | BD Biosciences | 563132 |
| CCR5 (CD195) | BV786 | 3A9 | BD Biosciences | 565001 |
| CXCR3 | FITC | G025H7 | Biolegend | 353704 |
| CD3 | BB700 | SP34-2 | BD Biosciences | 566517 |
| a4B7 | PE | A4B7R1 | NHPRR | AB_2819258 |
| CCR4 (CD194) | PE-CF594 | 1G1 | BD Biosciences | 565391 |
| CXCR5 (CD185) | PE-Cy7 | MU5UBEE | Thermofisher | 25-9185-42 |
| CD45 | BUV395 | D058-1283 | BD Biosciences | 564099 |
| CD8 | BUV496 | RPA-T8 | BD Biosciences | 612942 |
| CD69 | BUV737 | FN50 | BD Biosciences | 612817 |
| **Intracellular marker** | **Fluorophore** | **Clone** | **Company** | **Cat #** |
| Ki67 | AF700 | B56 | BD Biosciences | 561277 |

**Table S5:** T cell phenotype flow cytometry antibody panel for rectal biopsy.
